# Disrupting α-Synuclein–ClpP interaction restores mitochondrial function and attenuates neuropathology in Parkinson’s disease models

**DOI:** 10.1186/s13024-025-00918-w

**Published:** 2025-12-22

**Authors:** Di Hu, Xiaoyan Sun, Xin Qi

**Affiliations:** 1 Department of Physiology & Biophysics, Case Western Reserve University School of Medicine, Cleveland, OH 44106 USA; 2 Center for Mitochondrial Research and Therapeutics, Case Western Reserve University School of Medicine, 10900 Euclid Ave, E516, Cleveland, OH 44106-4970 USA

**Keywords:** ClpP, αSyn aggregation, Parkinson’s disease, Protein–protein interaction, Decoy peptide inhibitor, Mitochondrial proteostasis, Mitochondrial dysfunction

## Abstract

**Background:**

Mitochondrial dysfunction and α-Synuclein (αSyn) aggregation are defining features of Parkinson’s disease (PD), yet the mechanistic link between them remains poorly understood. Although our previous findings suggest that the interaction between αSyn and ClpP (a mitochondrial matrix protease) contributes to PD progression, the pathogenic and therapeutic relevance of this interaction remains elusive.

**Methods:**

We employed biochemical and cell biological approaches to investigate how αSyn and ClpP are mutually regulated. Additionally, we determined the pathogenic impact of αSyn–ClpP interaction by using decoy peptide CS2 in αSyn-PFF inoculated primary neurons, PD patient iPSC-derived dopaminergic neurons, and a transgenic mouse model of PD carrying αSyn-A53T mutation.

**Results:**

We identified mitochondrial protease ClpP as a key regulator of αSyn pathology. We show that αSyn interacts with ClpP through its non-amyloid-β component (NAC) domain, leading to impaired ClpP activity and mitochondrial proteotoxic stress. ClpP, in turn, negatively regulates αSyn aggregation and propagation by stabilizing its native tetrameric form. To interrupt this pathogenic interaction, we developed a decoy peptide, CS2, which binds the NAC domain of αSyn and restores ClpP function. CS2 treatment reduced mitochondrial oxidative stress and αSyn neurotoxicity in neuronal cultures, primary cortical neurons inoculated with αSyn preformed fibrils, and dopaminergic neurons derived from PD patient iPSCs. In mThy1-hSNCA transgenic mice, subcutaneous administration of CS2 restored ClpP levels, decreased αSyn pathology and neuroinflammation, and improved both cognitive and motor function.

**Conclusion:**

These findings highlight the αSyn–ClpP interaction as a druggable target and support CS2 as a potential disease-modifying therapy for PD and related synucleinopathies.

**Supplementary Information:**

The online version contains supplementary material available at 10.1186/s13024-025-00918-w.

## Introduction

Parkinson’s disease (PD) is the most common neurodegenerative movement disorder, affecting up to 2% of individuals aged 60 and older [1]. It is characterized by the progressive loss of dopaminergic (DA) neurons in the substantia nigra (SN) and the accumulation of cytoplasmic inclusions containing α-Synuclein (αSyn), known as Lewy bodies [2]. Despite significant advances in understanding PD pathophysiology, no effective treatments have been established to slow disease progression. This underscores the need to identify disease-driving mechanisms that can be targeted therapeutically.

Accumulating evidence indicates that mitochondria are primary targets of αSyn toxicity, and the interplay between αSyn and mitochondria may play a causal role in PD neuropathology [3, 4]. αSyn contains a cryptic mitochondrial targeting sequence and is enriched in mitochondria within vulnerable brain regions in PD [5]. Mitochondrial accumulation of αSyn induces bioenergetic deficits and increases reactive oxygen species (ROS) production, exacerbating αSyn toxicity, neuron degeneration, and associated cognitive and motor deficits [6, 7]. Within mitochondria, disturbances in protein homeostasis due to compromised protein quality control mechanisms exacerbate this toxicity, leading to proteostatic collapse, respiratory failure, and neuron loss in various PD models [8, 9].

The mitochondrial matrix protease ClpP plays a crucial role in maintaining mitochondrial proteostasis by processing unfolded or misfolded proteins for degradation, functioning similarly to a proteasome [10, 11]. We were first to show that pathological αSyn binds to ClpP, inhibiting its proteolytic activity and inducing mitochondrial misfolded protein accumulation and energetic failure [12]. Notably, genetic restoration of ClpP in PD patient induced pluripotent stem cells (iPSCs)-derived neurons or αSyn transgenic PD mice reduced αSyn pathology and improved animal behavioral outcomes [12]. These findings suggest that the αSyn–ClpP interaction contributes to disease progression and may represent a targetable pathway.

In this study, we investigated the pathogenic and therapeutic relevance of this interaction. We identified the non-amyloid-β component (NAC) domain of αSyn as the site of ClpP binding and found that this interaction promotes αSyn aggregation and mitochondrial stress. To block this pathogenic mechanism, we developed a rationally designed decoy peptide, CS2, which selectively targets the NAC domain and prevents αSyn from binding ClpP. CS2 restored ClpP function and mitigated αSyn-induced toxicity across several PD models, including cultured neurons, αSyn preformed fibril (PFF)-inoculated primary cortical neurons, and PD patient iPSCs-derived DA neurons. In αSyn transgenic mice (mThy1-hSNCA), CS2 treatment reduced αSyn aggregation and neuroinflammation and improved both motor and cognitive function. These findings identify the αSyn–ClpP interaction as disease-driving mechanism underlying αSyn-associated neurodegeneration and support the use of CS2-like peptides as a therapeutic strategy for PD and related synucleinopathies.

## Methods and materials

### Antibodies and reagents

Protein phosphatase inhibitor and protease inhibitor cocktails were purchased from MilliporeSigma. MitoTracker Orange CMTMRos (HY-D1696) and ClpP inhibitor A2-32-01 (HY-111532) were purchased from MedChemExpress. MitoTracker Red CMXRos (M7512) was purchased from ThermoFisher Scientific. For western blot, antibodies against ClpX (ab168338, 1:2000), ClpP (ab124822, 1:1000), αSyn (ab138501, 1:3000), and pS129-αSyn (ab168381, 1:1000) were from Abcam. Antibodies against GFP (sc-9996, 1:1000), c-Myc (sc-40, 1:1000), αSyn (sc-12767, 1:1000), were from Santa Cruz Biotechnology. Antibodies against Flag (F1804. 1:5000), β-Actin (A1978, 1:10000) were from MilliporeSigma. Antibodies against LONP1 (15440-1-AP, 1:3000) was from Proteintech. For immunostaining, antibodies against TH (MAB318, 1:1000), NeuN (ABN90, 1:500), and αSyn aggregates (clone 5G4) (MABN389, 1:500) were from MilliporeSigma. Antibody against TOM20 (11802-1-AP, 1:2000) was from Proteintech. Antibodies against Calnexin (ab22595), and pS129-αSyn (EP1536Y, 1:1000) were from Abcam. Antibodies against αSyn (Syn204) (2647, 1:500), and MAP2 (4542, 1:1000) were from Cell Signaling Technology. Antibody against Tuj1 (801201, 1:1000) was from Biologend. Antibody against Synapsin1 (106104, 1:500) was from SYSY Synaptic System. Antibody against PSD95 (MA1-045, 1:500) was from ThermoFisher Scientific. Antibody against ClpP (NBP1-89557, 1:200) was from Novus Biologicals. Antibody against TH (TYH0020, 1:1000) was from avesLab.

### Human recombinant αSyn species

The human recombinant WT- (#S-1001-2), A53T- (#S-1001-2), ΔNAC- (#S-1015-1) and ΔC- (#S-1012-1) αSyn and FITC-αSyn (S-1113-2) were purchased from rPeptide. The human recombinant αSyn pre-formed fibrils (PFF) (Type 1) (SPR-322) and ATTO-594-PFF (SPR-322B-A594) were purchased from StressMarq Biosciences (Bioactivity has been widely validated via various approaches, e.g. TEM, ThT).

### Decoy peptide synthesis

Decoy peptide CS1 (TAT-C6-GAVVTG) (Cat#: P1 YG-18, Lot#: P190717-MJ738690) and CS2 (TAT-C6-GGVVTA) (Cat# P2 YA-18, Lot#: P190717-MJ738691), TAT (# 976080), FITC-TAT (# 976082), FITC-CS2 (# 976081), Biotin-TAT (# P1601465), and Biotin-CS2 (# P201023) were synthesized at Ontores (Hangzhou, China). Their purities were assessed as > 90% by mass spectrometry. Lyophilized peptides were dissolved in sterile water and stored at -80 °C until use.

### Cell culture

SH-SY5Y cells were cultured in Dulbecco’s Modified Eagle Medium (DMEM)/F12 (1:1) supplemented with 10% (v/v) fetal bovine serum (FBS) and 1% (v/v) antibiotics (100 µg/ml penicillin and 100 µg/ml streptomycin) at 37℃ in a 5% CO_2_ incubator. HEK293T cells were cultured in DMEM supplemented with 10% (v/v) FBS and 1% (v/v) antibiotics.

### Preparation of total protein lysates

Cells or tissues were washed with 1X phosphate-buffered saline (PBS) and then incubated in total lysis buffer (10 mM HEPE-NaOH [pH 7.8], 150 mM NaCl, 1 mM ethylene glycol-bis [β-aminoethyl ether]-*N*,*N*,*N’*,*N’*-tetraacetic acid [EGTA], 1% Triton X-100, protease inhibitors, and phosphatase inhibitors) for 30 min on ice. Samples were collected and centrifuged at 12,000 rotations per minute for 10 min at 4 °C. The supernatants were saved as total lysates.

### Isolation of mitochondrial fractions

Cells were washed with cold PBS and incubated on ice for 30 min in a lysis buffer (250mM sucrose, 20mM HEPES-NaOH, pH 7.5, 10mM KCl, 1.5mM MgCl2, 1mM EDTA, protease inhibitor cocktail and phosphatase inhibitor cocktail). Mouse brains were minced and homogenized in the lysis buffer and then placed on ice for 30 min. Collected cells or tissue were disrupted 20 times by repeated aspiration through a 25-gauge needle, followed by a 30-gauge needle. The homogenates were spun at 800 g for 10 min at 4 °C, and the resulting supernatants were spun at 10,000 g for 20 min at 4 °C. The pellets were washed with lysis buffer and spun at 10,000 g again for 20 min at 4 °C. The final pellets were suspended in lysis buffer containing 1% Triton X-100 and were mitochondrial-rich lysate fraction. Mitochondrial fractions were further digested by 50 µg/ml proteinase K for 30 min at room temperature, which was terminated by adding 5 mM PMSF.

### Preparation of triton-soluble and -insoluble fraction

Tissues were homogenized and incubated on ice for 30 min in total lysis buffer (50 mM Tris–HCl, pH 7.5, 150 mM NaCl, 1% Triton X-100, and protease inhibitor). Tissues were then spun at 12,000 rpm for 10 min at 4 °C; the resulting supernatant was triton-soluble fraction. The pellet was further suspended in total lysis buffer with 1% SDS, sonicated and incubated at 100 °C for 3 min, and followed by 20-s vortex. After two more repeats of 100 °C incubation and vortex, the solution was spun at 12,000 rpm for 10 min at 4 °C; the resulting supernatant was triton-insoluble fraction.

### Preparation of digitonin-soluble and insoluble fraction

Cells were washed with cold PBS and incubated on ice for 30 min in lysis buffer (50mM Tris pH 7.4, 150mM NaCl, protease inhibitor cocktail) containing 0.5% or 1% digitonin. Mouse brains were minced and homogenized in the lysis buffer and then placed on ice for 30 min. Collected cells or tissue were spun at 20,000 g for 20 min at 4 °C, the result supernatant was digitonin soluble fraction. The pellets were further suspended in digitonin lysis buffer with 1% SDS, and incubated at 100 °C for 3 min, followed by 20s vortex. After two more repeats of 100 °C incubation and vortex, the solution was spun at 20,000 g for 20 min at 4 °C, the result supernatant was digitonin insoluble fraction.

### Western blot

Protein concentrations were measured using the Bradford assay. Proteins (30 µg) were then resuspended in Laemmni buffer, loaded on sodium dodecyl sulfate-polyacrylamide gel electrophoresis gels, and transferred to nitrocellulose membranes. The membranes were probed with the indicated antibodies and visualized by electrochemiluminescence.

### Constructs and transfections

EGFP-tagged WT αSyn (#40822), Venus-N-αSyn (#89470) and αSyn-C-Venus (#89471) plasmids were purchased from Addgene. Myc-tagged WT- and A53T- αSyn, Myc-tagged ClpP, and Flag-tagged WT- and S153A- ClpP plasmids were self-constructed as previously described (Ref). To construct truncated-αSyn plasmids, pcDNA3.1(-) was digested with BsteII and XbaI, and ΔN-, ΔC- and ΔNAC-αSyn was PCR-amplified using self-designed primers and inserted into the plasmid backbone. Cells were transfected with *Trans*IT-2020 (Mirus Bio, LLC) following the manufacturer’s protocol.

### Microscale thermophoresis

MST experiments to determine CS2 and FITC-synuclein binding affinity (*KD*) were performed as described in detail elsewhere (13). Briefly, CS2 peptide (ligand) was diluted to 16 different concentrations ranging from 2.24 nM to 80 µM using buffer containing 20 mM Tris pH 7.4 and 100 mM NaCl. Each ligand sample was mixed with an equal volume of 0.6 µM FITC-αSyn. After 15 min incubation at room temperature, the samples (16 dilutions of liposomes with constant protein) were loaded into 16 Monolith NT.115 Premium Treated Capillaries (NanoTemper Technologies) and MST was measured at 25 °C using the Monolith NT.115 instrument (NanoTemper Technologies). Instrument parameters were adjusted to 20% LED power and high MST power. For *KD* analysis, ligand-dependent changes in MST are plotted as fraction bound versus ligand concentration in a dose-response curve. Data from three independent measurements were analyzed using the signal from the MST-off time of -1.0–0.0s and MST-on time of 1.5–2.5 s (MO.Affinity Analysis software version 2.2.7, NanoTemper Technologies) and plotted using Origin^®^ statistical software.

### Peptides treatment in αSyn PFF-inoculated primary cortical neurons

Primary cortical neurons were isolated from normal E18 mouse cortex (sex undetermined) and cultured using complete culturing kit (cat#: KTC57ECX, TransnetYX Tissue). Briefly, 100,000 cells were seeded on poly-D-lysine coated glass coverslips (PDL15mm, TransnetYX Tissue) and cultured in NbActiv1 medium (NB1, TransnetYX Tissue). Neurons were treated with sonicated human α-Syn-PFF (SPR-322, StressMarq Biosciences) starting on post-seeding Day 5 till Day 14. Starting from post-seeding Day 9, TAT or CS2 peptide was added daily at 1µM. Neurons were fixed on Day 14 and subjected for experiments.

### Peptides administration in mThy1-hSNCA mice

All animal experiments were conducted in accordance with protocols approved by the Institutional Animal Care and Use Committee of Case Western Reserve University and were performed based on the National Institutes of Health Guide for the Care and Use of Laboratory Animals. Sufficient procedures were employed for reduction of pain or discomfort of subjects during the experiments.

C57BL/6 N-Tg (Thy1-SNCA)15Mjff/J (JAX: 017682) breeders (C57Bl/6NJ genetic background) were purchased from Jackson Laboratories. The mice were mated, bred, and genotyped in the animal facility of Case Western Reserve University. All mice were maintained at a 12-hour light/dark cycle (on 6 am, off 6 pm). All randomization and peptide treatments in mThy1-hSNCA mice were prepared by an experimenter not associated with the behavioral and neuropathology analysis. Only male mice were used in the study. mThy1-hSNCA transgenic mice and their age-matched WT littermates were implanted with a 28-day subcutaneous osmotic pump (Alzet, Cupertino CA, Model 2004) containing either TAT control peptide or CS2 peptide, which delivered the peptides at indicated rate (1 or 3 or 10 mg/kg/day). The pump was replaced once every four weeks. All mice were sacrificed at age of 10-month for neuropathology assessment.

### Behavioral analysis

All behavioral analyses were conducted by an experimenter who was blinded to the genotypes and treatment groups. All mice were subjected to a series of behavioral measurements to monitor locomotor activity (open field test), spontaneous spatial working memory (Y-maze test), and motor coordination (Rotarod test).

#### Y-maze test

On the test day, mice at 6-month-old were brought to the testing room one hour before performing the Y-maze test to allow habituation. The mice were placed in the middle of the Y-maze and allowed to explore the three arms for 4-min. During exploration, the arm entries were recorded. The equipment was cleaned after every test to avoid odor disturbance. Spontaneous alternation was defined as a successive entry into three different arms on overlapping triplet sets.

#### Open field chamber

The locomotor activity of all experimental mice was assessed in an open field chamber at 8-month-old. Briefly, the mice were placed in the center of an activity chamber (Omnitech Electronics) and allowed to explore the chamber while being tracked using an automated infrared tracking system (Vertax, Omnitech Electronics). A 24-hour locomotor activity analysis was performed. Both horizontal and vertical activity/movements were recorded.

#### Rotarod test

Mice were trained for the accelerating test on Day 1–3. On Day 5, motor coordination was assessed in TAT or CS2 treated mThy1-hSNCA transgenic mice and their age-matched wild-type littermates at age of 10-month, by measuring latency on a rotarod in an accelerated program for 300 s. Body weights of mThy1-hSNCA mice and wild-type littermates were recorded throughout the study period.

### Peptides treatment in human iPSC-derived neurons

PD iPS cells lines (αSyn A53T, NN0004337, female donor) and its isogenic corrected control line (NN0004344) were obtained from RUCDR Infinite Biologics. The iPS cells were differentiated into dopaminergic neuron-enriched neuronal culture with the protocol described previously [13, 14]. Briefly, iPS cell colonies were disassociated with accutase (Invitrogen), plated onto 6-well plates pre-coated with 2.5% Matrigel (BD Biosciences) and allowed to reach 80% confluence in mTeSR medium (Stem Cell Technology). For the first 10 days, cells were treated with SB431542 (10 µM; Tocris Bioscience) and Noggin (100 ng/ml) in Neural Media (NM) with FGF2 (20 ng/µl) and EGF (20 ng/µl). NM media contained: Neurobasal and DMEM/F12 (1:1), B-27 Supplement Minus Vitamin A (50×, Invitrogen), N2 Supplement (100X, Invitrogen), GlutaMAX (Invitrogen, 100×), 100 units/ml penicillin and 100 µg/ml streptomycin (Fisher); for the next 4 days, cells were treated with human recombinant Sonic hedgehog (SHH, 200 ng/ml) in neuronal differentiation medium. Neuronal differentiation medium contained Neurobasal and DMEM/F12 (1:3), B27, N2, GlutaMax and PS. In the following 3 days, cells were switched to BDNF (20 ng/ml), ascorbic acid (200 µM, Sigma-Alderich), SHH (200 ng/ml), and FGF8b (100 ng/ml) in neuronal differentiation medium. Thereafter, cells were treated with BDNF, ascorbic acid, GDNF (10 ng/ml), TGF-b (1 ng/ml), and cAMP (500 M, Sigma-Aldrich). All growth factors were purchased from Pepro Tech (Rocky Hill, NJ, USA). After 20 days of induction, neurons were treated daily with TAT or CS2 peptides at 1µM and were fixed for immunostaining at 26 days of induction. The imaging was observed by confocal microscope (Fluoview FV3000, Olympus).

### Intact cell crosslinking to detect αSyn tetramer

After washing with PBS (pH 8.0), cell pellets were incubated in 1mM DSG solved in PBS for 40 min at 37 °C with 650 rpm shaking on Eppendorf ThermoMixer C. The reaction was then quenched with 50mM Tris pH 7.4 for 15 min at room temperature. The cells were then lysed by ultrasonication for 15s at 20% amplitude (Branson Digital Sonifier). Cells lysates were then centrifuged at 20,000 g at 4 °C for 40 min. The supernatant was then collected for western blot analysis. αSyn monomer and oligomers can be detected using anti-αSyn (sc-12767, 1:1000) antibody from Santa cruz Biotechnology.

### αSyn ThT assays

αSyn monomer (5µM) and PFF (1µM) were incubated with recombinant ClpP (5/10µM) and Thioflavin T (ThT) (1µM) in 100 µl PBS (pH 7.4) at 37 °C in a shaking incubator (500 rpm). ThT fluorescence was measured (excitation 450 nm, emission 485 nm) every 15 min up to 48 h.

### Immunofluorescence staining

Cells were grown on coverslips, fixed with 4% paraformaldehyde for 20 min at room temperature, permeabilized with 0.1% Triton X-100 in PBS, and blocked with 2% normal goat serum. The cells were incubated with the indicated primary antibodies overnight at 4℃. After washing with PBS, the cells were incubated with Alexa Fluor 488/568 or 405/568 secondary antibody (1:500; Thermo Fisher Scientific) for 2 h at room temperature. The nuclei were counterstained with DAPI (1:10000; Sigma-Aldrich). Images of the staining were acquired using a Fluoview FV3000 confocal microscope (Olympus).

For immunofluorescence staining of mouse brain sections, mice were deeply anesthetized and transcardially perfused with 4% paraformaldehyde in PBS. Brain sections were washed in PBS 3 times and subjected for antigen retrieval in 0.01 M sodium citrate buffer with 0.05% TWEEN-20, followed by permeation with 0.2% Triton X-100 in PBS at room temperature for 10 min. Sections were then blocked with 10% normal goat serum in PBS and incubated with primary antibodies overnight at 4 °C and then stained with secondary antibodies. Images of the staining were acquired using Fluoview (FV3000) confocal microscope (Olympus).

All quantification of immunostaining was performed using Fiji ImageJ software. The same image exposure times and threshold settings were used for all sections from all the experimental groups. Quantitation was performed blinded to the experimental groups. 

### Proximity ligation assay

Proximity ligation assay (PLA) was performed using the Duolink® In Situ Starter Kit with Detection Reagents Green (mouse/rabbit; DUO92101, DUO92014). Neurons were stained with MitoTracker Orange CMTMRos for 20 min, fixed in 4% paraformaldehyde, and blocked in Duolink blocking buffer for 1 h at 37 °C. Cells were then incubated overnight at 4 °C with anti-ClpP (66271-1-Ig; Proteintech) and anti-α-synuclein (ab138501; Abcam) primary antibodies. After washing, samples were incubated with PLA probes (anti-rabbit PLUS and anti-mouse MINUS) for 1 h at 37 °C, followed by ligation and amplification according to the manufacturer’s instructions. PLA puncta were detected as distinct fluorescent spots and imaged by confocal microscopy (Fluoview FV3000; Olympus). 

### Immunohistochemistry

Frozen brain sections (20 μm, coronal) were washed in PBS 3 times and subjected for antigen retrieval in 0.01 M sodium citrate buffer with 0.05% TWEEN-20. After washing 3 times in TBS, sections were then incubated in 3%H2O2/w methanol for 30 min at room temperature to quench endogenous peroxidase. After washing in TBS, sections were blocked in 10% normal goat serum with 1XTBST for one hour at room temperature and incubated with anti-pS129-αSyn (ab51253, clone EP1536Y, abcam) or anti- αSyn aggregates (MABN389, clone 5G4, Millipore) antibody in TBST overnight. Secondary antibody and DAB staining were provided by the IHC Select HRP/DAB kit (DAB150, Millipore). Hematoxylin QS (H-3404, Vector laboratory) was used to stain nucleus. The intensity of αSyn was conducted using Fiji ImageJ software. The same image exposure times and threshold settings were used for all sections from all treatment groups. Quantitation was performed blinded to the experimental groups.

### In vitro ClpP peptidase activity

ClpP peptidase activity in vitro was measured as previously described (REF). Briefly, human recombinant ClpP (10 µM, obtained from Dr. Aaron Schimmer, Princes Margartet Cancer Centre, Canada) were incubated in the reaction buffer (50mM Tris PH 8.0, 200mM KCl, 1mM DTT, 2mM ATP) under 37 °C for 10 min. For co-incubation with αSyn, recombinant ClpP and WT or C-terminal truncated or NAC-domain truncated recombinant αSyn (S-1001-1, S-1012-1, S-1015-1, rPeptide) were pre-incubated for 30 min under room temperature. Fluorescent substrate of ClpP, ac-WLA-AMC (50 µM), were then added in the reaction buffer. The fluorescence signal was read using TECAN infinite M1000 up for 30 min at excitation/emission wavelength 345/445. ClpP peptidase activity were determined as the slope of the regression line.

### Co-immunoprecipitation

Cells or tissues were lysed in total lysis buffer (50mM Tris-HCl, pH 7.5, 150mM NaCl, 1% Triton X-100, and protease inhibitor). Total protein lysates (800 µg) were incubated with the indicated antibodies overnight at 4 °C followed by the addition of protein A/G beads (sc-2003, Santa Cruz) for 2 h at 4 °C. Various recombinant αSyn and Clpp (500 ng) were incubated in in vitro interaction buffer (20mM Tris-HCl pH 7.5, 100mM KCl, 2mM MgCl_2_ and 0.1% Triton-X100) for 30 min at room temperature, and then incubated with indicated antibodies overnight at 4 °C followed by the addition of protein A/G beads for 2 h at 4 °C. The beads with immuno-precipitates were pulled down at 4000 g and washed four times with total lysis buffer/in vitro interaction buffer in the presence of 0.1% Triton X-100 and were analyzed by western blot analysis.

### Mitochondrial ROS measurement

Cells cultured on coverslips or 24-well plates were washed with DPBS and then incubated with 5 µM Mito-SOX™ Red (Invitrogen, M36008), a mitochondrial superoxide indicator, for 10 min at 37 °C. For cells cultured on coverslips, the images were visualized by microscope, and quantification of images was then carried out using NIH ImageJ software. At least 100 cells per group were counted in the analysis.

### Mitochondrial membrane potential

Cells cultured on coverslips or 24-well plates were washed with DPBS and then incubated with 0.25 µM Tetramethylrhodamine, methyl ester (TMRM) (Invitrogen, T668), for 20 min at 37 °C. For cells cultured on coverslips, the images were visualized by microscope, and quantification of images was then carried out using NIH Fiji ImageJ software. At least 100 cells per group were counted in the analysis.

### Cytotoxicity measurement by LDH

SH-SY5Y cells were treated with different doses of TAT/CS2 peptides for 3 days, and cell death was determined using the Cytotoxicity Detection Kit (LDH), according to the manufacturer’s protocol (Roche, REF 11 644 793 001).

### Cellular ATP measurement

Cells were seeded in 24-well plates, followed by the indicated treatment. The ATP level in each well was determined using the ATP assay kit according to the manufacturer’s procedures (Cayman Chemical, 700410).

### αSyn ELISA

After one day incubation with doxycycline (1µM), αSyn tet-on inducible expression SHSY5Y cells were treated with TAT or CS2 peptides at 10 µM for another two days. The level of αSyn monomer released into culture medium was measured using LEGEND MAX human αSyn ELISA kit (448607, Biolegend). The level of extracellular and intracellular αSyn oligomers was measured using human alpha synuclein oligomer ELISA kit (MBS730762, Mybioresource).

### Quantification and statistical analysis

Sample sizes were determined by a power analysis based on pilot data collected in our laboratory or from published studies. For animal studies, we used *n* = 10–15 mice/group for behavioral tests, *n* = 3–6 mice/group for biochemical analyses, and *n* = 3–10 mice/group for pathology studies. In cell culture studies, each experiment was independently conducted at least three times. For animal studies, we ensured randomization and blind evaluations. For imaging studies, a blind observer performed quantification analyses.

Data were analyzed using GraphPad Prism 10 (GraphPad Software, San Diego, CA, USA). Unpaired Student’s t test was used for comparisons between two groups. Comparisons between three or more independent groups were performed using one-way ANOVA, followed by Tukey’s post hoc test. Comparisons of the effect of independent variables on a response variable were performed using two-way ANOVA. All values are reported as mean ± standard error of the mean (SEM). Data are representative of at least three independent experiments. Statistical parameters are presented in each figure legend. We considered *p* < 0.05 as statistically significant.

## Results

### ClpP regulates αSyn tetramer stability, aggregation and propagation

**Fig. 1:**
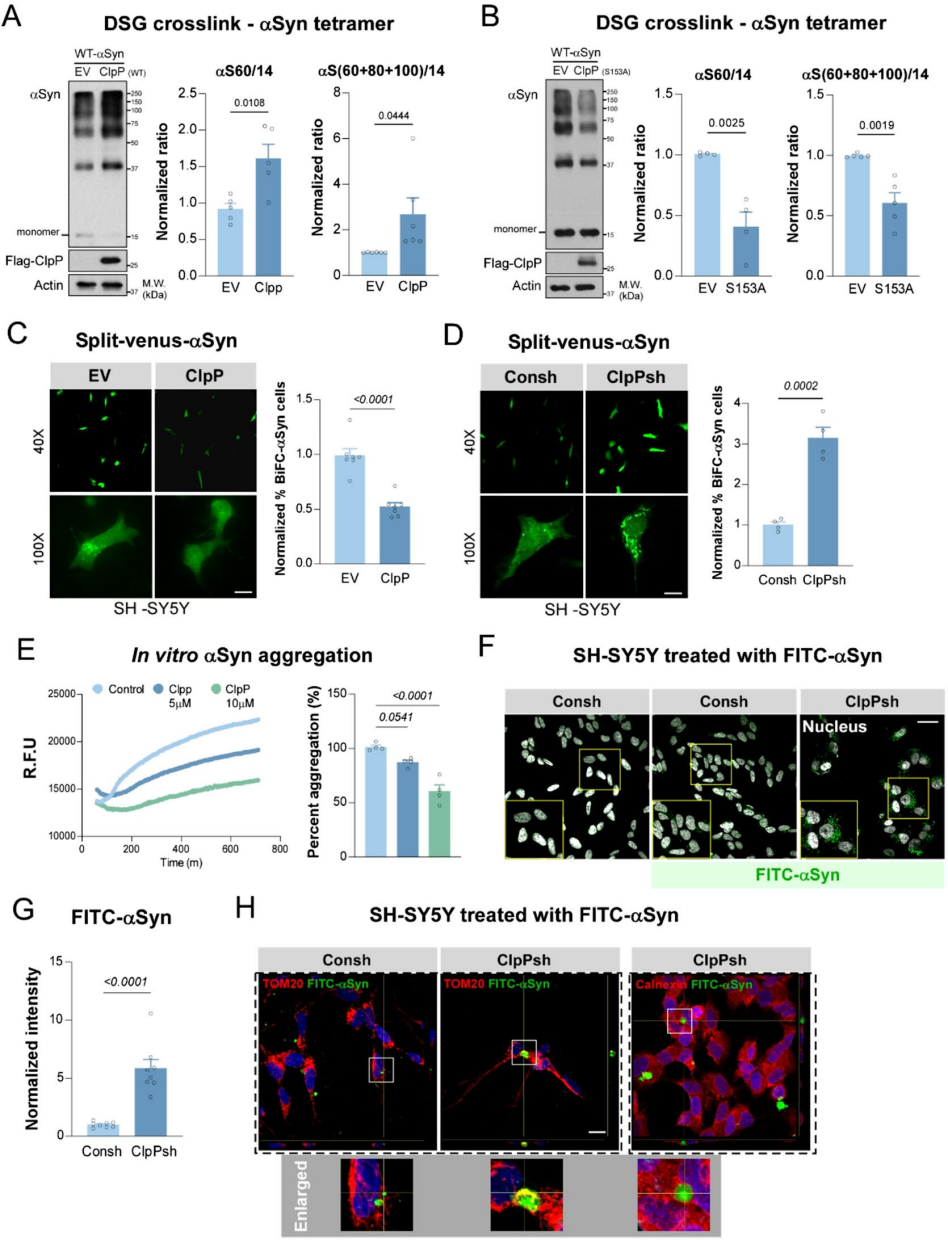
ClpP modulates αSyn tetramer and aggregates. (**A**) Western blot and quantification of αSyn tetramer/monomer ratio and oligomer/monomer ratio, upon intact cell DSG-crosslinking, in HEK293T cells overexpressing Myc-tagged wild-type (WT) αSyn with vector (EV) or Flag-tagged WT-ClpP (*n* = 5–6, two-tailed Student’s t test; data are mean ± SEM). (**B**) Western blot and quantification of αSyn tetramer/monomer ratio and oligomer/monomer ratio, upon intact cell DSG-crosslinking, in HEK293T cells overexpressing Myc-tagged WT αSyn with EV or Flag-tagged S153A-ClpP (*n* = 4–5, two-tailed Student’s t test). (**C**) Fluorescent images and quantification of αSyn aggregation in SH-SY5Y cells overexpressing EV or ClpP with vectors expressing Venus-N-αSyn (VN) and αSyn-C-Venus (VC) (*n* = 7, two-tailed Student’s t test, Scale bar = 10 μm). (**D**) Fluorescent images and quantification of αSyn aggregation in control knock-down (Consh) or ClpP knock-down (ClpPsh) SH-SY5Y cells overexpressing VN and VC (*n* = 4, two-tailed Student’s t test, Scale bar = 10 μm). (**E**) In vitro αSyn PFF (1µM) – seeded aggregation in the absence (control) or presence of ClpP (5/10µM) was monitored by thioflavin T (ThT) fluorescence (*n* = 4, two-tailed Student’s t test). (**F-G**) Representative images and quantification of FITC-αSyn in Consh or ClpPsh SH-SY5Y cells after 24-hour treatment with FITC-conjugated αSyn monomer (1µM) (Data are mean ± SEM, *n* = 8, two-tailed Student’s t test, scale bar = 30 μm; enlarged area were circled in yellow). (**H**) Representative images of TOM20 or Calnexin staining with FITC-αSyn in Consh or ClpPsh SH-SY5Y cells (scale bar = 20 μm; enlarged area are circled)

Physiological αSyn primarily exists as a tetramer, a conformation resistant to aggregation. However, under pathological conditions such as mitochondrial or lysosomal stress, this tetrameric structure can destabilize, shifting αSyn toward a monomeric state that is prone to oligomerization and fibril formation [15–17]. In HEK293T cells expressing wild-type αSyn (WT-αSyn), overexpression of bioactive ClpP significantly elevated both αSyn tetramer-to-monomer and oligomer-to-monomer ratios (Fig. 1A). In contrast, expression of a proteolytically inactive mutant ClpP (ClpP-S153A) led to a significant reduction in both ratios (Fig. 1B), suggesting that ClpP protease activity is required for stabilizing the tetrameric form of αSyn. Consistently, in dopaminergic SH-SY5Y neuronal cells, overexpression of ClpP elevated the endogenous αSyn tetramer-to-monomer ratio, whereas ClpP knockdown decreased it, without altering total αSyn levels (Fig. S1A–D). Notably, a reduced endogenous αSyn tetramer-to-monomer ratio can be also observed in the iPSC-derived neurons treated with A2-32-01 (Fig. S1E), a selective ClpP inhibitor [18]. Together, these findings support a model in which ClpP preserves αSyn in its native, aggregation-resistant tetrameric state and thereby constrains its transition to pathogenic forms.

We next examined whether ClpP influences αSyn aggregation and propagation, two pathological processes that are amplified following tetramer destabilization [16, 19, 20]. To assess αSyn oligomerization in living cells, we employed a bimolecular fluorescence complementation (BiFC) system using Venus-N-terminal and C-terminal tagged αSyn (VN/VC-αSyn) constructs [21]. In SH-SY5Y cells, ClpP overexpression led to a significant reduction in BiFC signal intensity (Fig. 1C), indicating decreased αSyn oligomer formation. Conversely, ClpP knockdown using shRNA resulted in a marked increase in BiFC-αSyn signal (Fig. 1D), consistent with enhanced oligomerization. To further determine whether ClpP modulates αSyn fibrillization, we conducted an in vitro seeded aggregation assay using αSyn PFFs and monitored aggregation by Thioflavin T (ThT) fluorescence probe [22, 23]. Incubation with recombinant ClpP protein led to a dose-dependent suppression of PFF-ThT fluorescence intensity (Fig. 1E), suggesting that ClpP can directly inhibit fibril formation. We next assessed whether ClpP affects αSyn cell-to-cell propagation. SH-SY5Y cells with ClpP knockdown by shRNA (ClpPsh) or control shRNA (Consh) were treated with FITC-labeled αSyn (FITC-αSyn) to monitor internalization and intracellular aggregation. ClpP-deficient cells exhibited greater accumulation and aggregation of FITC-αSyn (Fig. 1F, G), indicating that ClpP deficiency facilitates αSyn uptake and intracellular seeding.

A recent study utilizing Cryo-electron tomography (Cryo-ET) and transmission electron microscope (TEM) implicates mitochondria as a preferential platform for αSyn accumulation and aggregation [24]. Consistent with this evidence, in the ClpP-deficient cells, FITC-αSyn formed detergent-resistant puncta that colocalized with the mitochondrial marker TOM20 (Fig. 1H) and MitoTracker Red (Fig. S1F) but not with calnexin (an endoplasmic reticulum protein) (Fig. 1H). Compared with control cells, FITC-αSyn level was significantly higher in proteinase K-digested mitochondrial fraction from ClpP-deficient cells (Fig. S1G). These findings suggest that in the absence of ClpP, αSyn preferentially accumulates and aggregates within mitochondria.

Together, our results demonstrate that ClpP suppresses αSyn oligomerization, aggregation, and propagation, likely by maintaining αSyn in its non-pathogenic tetrameric form and limiting its mitochondrial accumulation.

### ClpP interacts with αSyn NAC domain

**Fig. 2:**
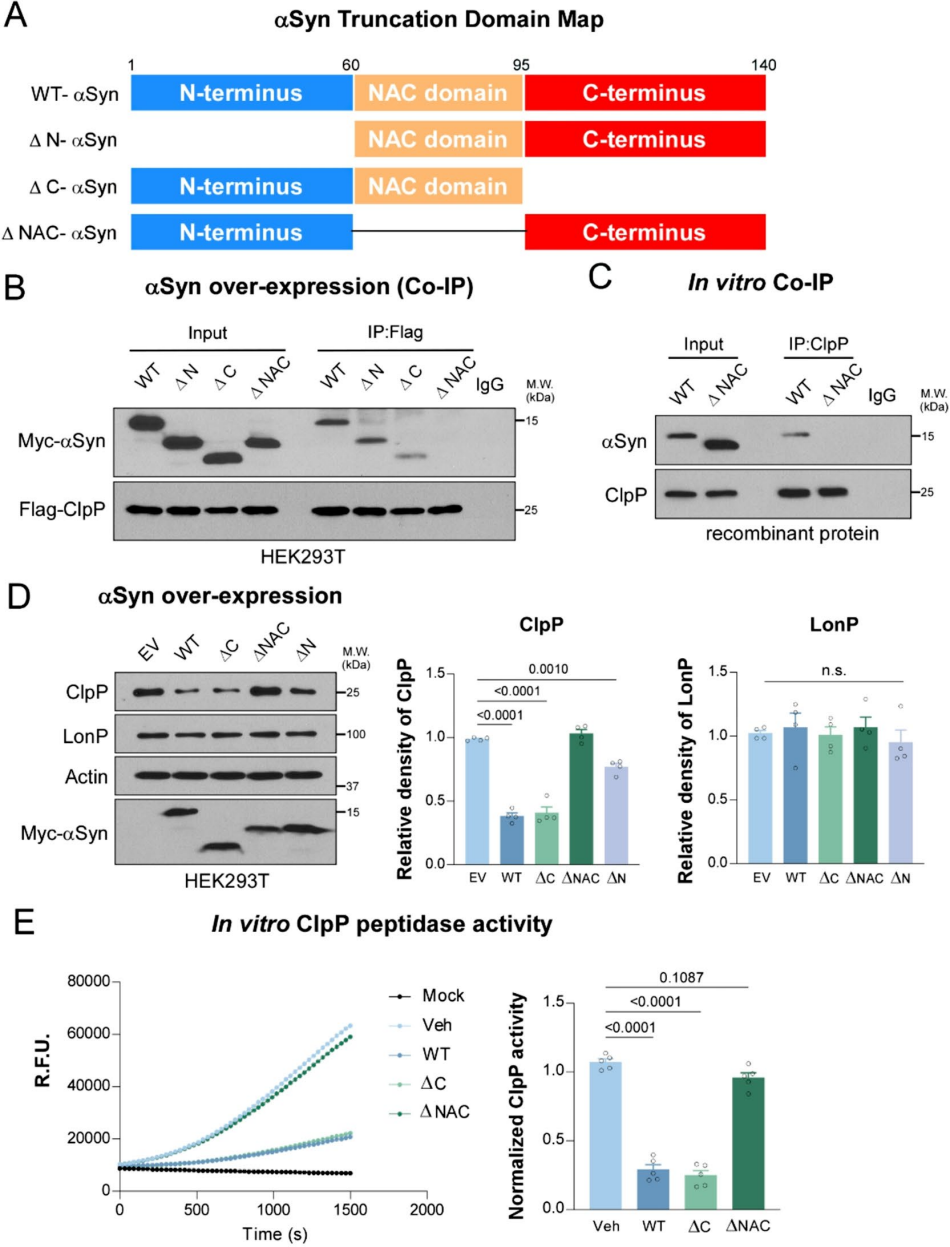
αSyn NAC domain interacts with ClpP. (**A**) Domain map of wild-type (WT)-αSyn and N-terminal truncated (ΔN), C-terminal truncated (ΔC), or NAC-domain truncated (ΔNAC) αSyn. (**B**) Total lysates were harvested from HEK293T cells overexpressing Myc-tagged WT-, ΔN-, ΔC-, or ΔNAC-αSyn with Flag-tagged ClpP and subjected for immunoprecipitation using anti-Flag antibody, followed by western blot analyses. Shown are the representative blots of 3 independent experiments. (**C**) Recombinant ClpP was incubated with WT- or ΔNAC- αSyn for 12-hour, and pulled down using anti-ClpP antibody, followed by western blot analyses. Show are the representative blots of 3 independent experiments. (**D**) Western blot and quantification of ClpP and LonP in HEK293T cells overexpressing Myc-tagged WT-, ΔN-, ΔC-, or ΔNAC-αSyn (*n* = 4, one-way ANOVA with Tukey’s *post-hoc* test). (**E**) In vitro ClpP peptidase activity was measured in the absence (Veh) or presence of WT-, ΔC-, or ΔNAC-αSyn. The fluorescence intensity of ac-WLA-AMC (50 µM), a fluorogenic substrate of ClpP, was measured (0–30 min) immediately after addition (*n* = 5, one-way ANOVA with Tukey’s *post-hoc* test; data are mean ± SEM)

To gain mechanistic insights into the interaction between ClpP and αSyn, we examined which protein domain of αSyn binds to ClpP and contributes to the suppression of ClpP proteolytic activity. ClpP is a mitochondrial matrix protease composed of conserved α-helical domains organized into a barrel-like structure, as revealed by X-ray crystallography and cryo-electron microscopy [25]. In contrast, αSyn is a natively unfolded protein comprising three functional regions: an N-terminal domain, the NAC domain, and a C-terminal domain. Among these, the NAC domain is known to drive β-sheet formation and amyloid fibril assembly during αSyn aggregation [26]. To map the ClpP-interacting region within αSyn, we generated a series of Myc-tagged αSyn truncation constructs lacking the N-terminal (ΔN), C-terminal (ΔC), or NAC (ΔNAC) domains (Fig. 2A). These constructs were co-expressed in HEK293T cells with Flag-tagged ClpP, and their interactions were assessed by co-immunoprecipitation (Co-IP). WT-αSyn, as well as the ΔN and ΔC mutants, robustly co-precipitated with ClpP, whereas the ΔNAC mutant failed to bind (Fig. 2B). This domain-specific interaction was further confirmed in vitro using recombinant proteins. While WT-αSyn protein interacted with ClpP protein, ΔNAC-αSyn protein did not (Fig. 2C). Thus, the NAC domain is required for direct binding of αSyn to ClpP.

We next asked whether the NAC domain is also required for αSyn-mediated suppression of ClpP. In HEK293T cells, overexpression of WT-, ΔN-, or ΔC-αSyn significantly reduced ClpP protein levels (Fig. 2D). In contrast, expression of ΔNAC-αSyn had little to no effect (Fig. 2D). Moreover, in in vitro peptidase assays, recombinant WT-αSyn and ΔC-αSyn greatly impaired ClpP activity, while the ΔNAC mutant showed negligible inhibition (Fig. 2E). Together, these findings demonstrate that the NAC domain of αSyn is both necessary and sufficient for its interaction with ClpP and for the subsequent suppression of ClpP protease function. This interaction provides a mechanistic link between αSyn aggregation propensity and mitochondrial proteostasis disruption in PD.

### A decoy peptide CS2 disrupts ClpP-αSyn interaction and restores ClpP activity

To determine the direct impact of αSyn-ClpP interaction on mitochondrial proteostasis and αSyn neuropathology, we propose the use of rationally designed peptide inhibitors to interfere with ClpP and αSyn interaction. Two non-related proteins that interact in an inducible manner often have shared short sequences of homology that could represent binding sites of both inter- and intra-molecular interactions [27–30]. A peptide corresponding to that sequence can serve as decoys for one of the proteins, preventing the binding of that protein to the target protein [27–29, 31]. Our lab has pioneered the use of rationally designed short peptides to interfere with protein-protein interactions inside cells related to VCP and mtHtt [32], Drp1 and Fis1 [33], and Drp1 and ATAD3A [34]. We used these peptide regulators as a pharmacological tool to identify the role of these proteins in mitochondrial signal transduction in neurodegenerative diseases such as Huntington’s disease (HD) [32, 35] and Alzheimer’s Disease (AD) [34, 36] and other disease models [14, 37–41].

**Fig. 3:**
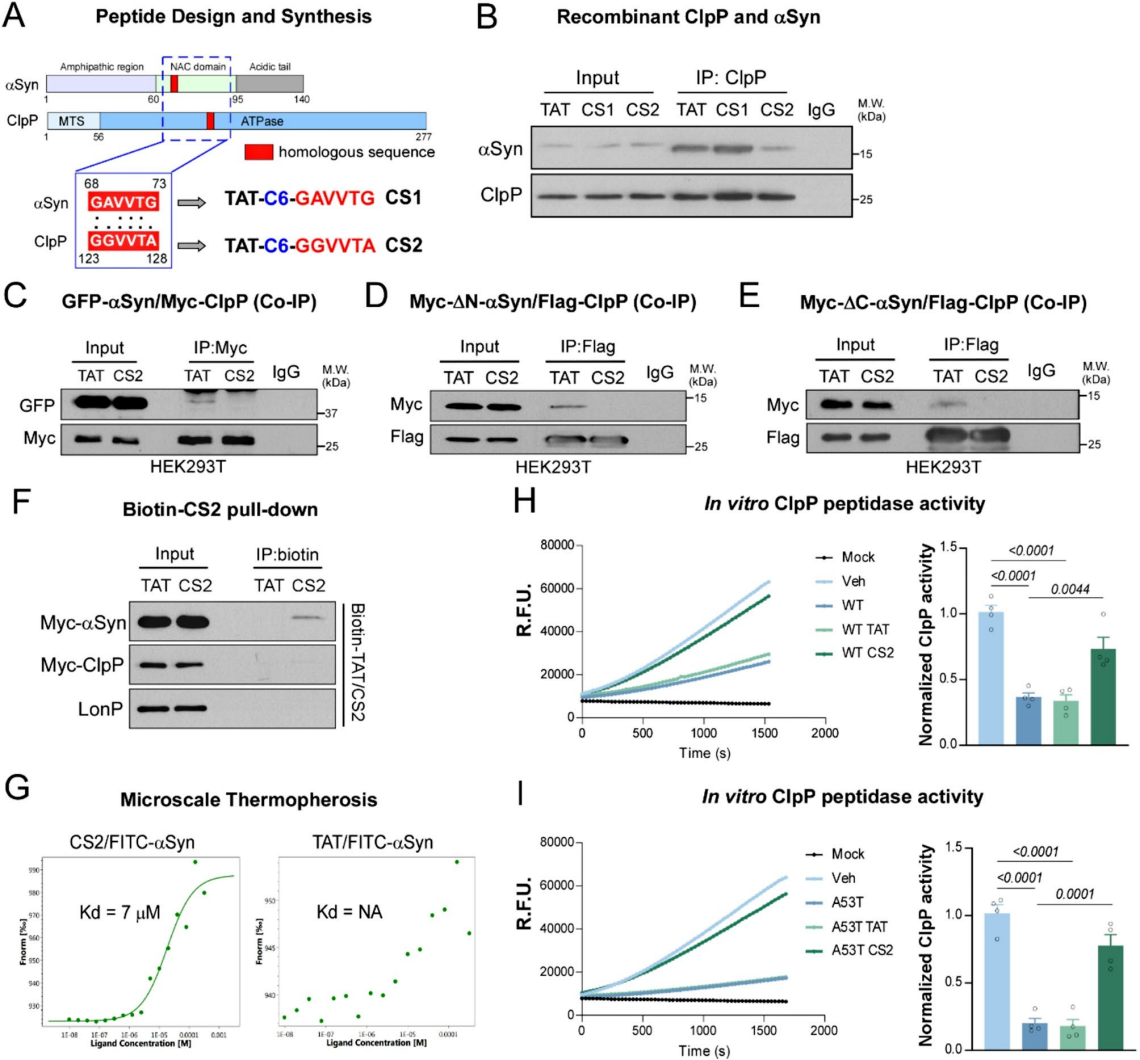
Decoy peptide CS2 disrupts the interaction between ClpP and αSyn. (**A**) Design schematic of decoy peptides CS1 and CS2. CS1 - TAT sequence is linked to N-terminus of the homologous sequence on αSyn by C6; CS2 – TAT sequence is linked to N-terminus of the homologous sequence on ClpP by C6. (**B**) Recombinant ClpP was pre-incubated with control peptide TAT, CS1, or CS2 for 30 min, before incubation with αSyn, and subjected for immunoprecipitation using anti-ClpP antibody, followed by western blot analyses. Shown are representative blots of 3 independent experiments. (**C**) Total lysates were harvested from HEK239T cells overexpressing GFP-tagged αSyn with Myc-tagged ClpP after 24-hour treatment with TAT or CS2 peptides, and subjected for immunoprecipitation using anti-Myc antibody, followed by western blot analyses. Shown are representative blots of 3 independent experiments. (**D**-**E**) Total lysates were harvested from HEK293T cells overexpressing Flag-tagged ClpP with Myc-tagged ΔN- or ΔC-αSyn, and subjected for immunoprecipitation using anti-Flag antibody, followed by western blot analyses. Shown are representative blots of 3 independent experiments. (**F**) Total lysates were harvested from HEK293T cells overexpressing Myc-tagged αSyn or ClpP and incubated with Biotin-conjugated TAT or CS2 peptide for 12-hour. The Biotin-TAT or CS2 was pulled down by streptavidin-beads and subjected for western blot analyses. Shown are the representative blots of 3 independent experiments. (**G**) The binding affinity (Kd = 7µM) of CS2 with FITC-αSyn was determined by Microscale Thermophoresis (details see methods). Shown are representative curves of 3 independent experiments. (**H**-**I**) ClpP peptidase activity was measured after incubation with WT or mutant A53T αSyn in the presence of TAT or CS2 peptide. The fluorescence intensity of ac-WLA-AMC (50 µM), a fluorogenic substrate of ClpP, was measured (0–30 min) immediately after addition. Shown is the representative of 4 independent experiments (one-way ANOVA with Tukey’s *post-hoc* test; data are mean ± SEM)

Similar to the peptide designs for P110 [33], HV-3 [32] or DA1 [34], we used L-ALIGN sequence alignment software [42] and identified two regions of homology between human αSyn and human ClpP (Fig. 3A). The two regions are marked as CS1 and CS2 (Fig. 3A). We predicted that these peptides, corresponding to sequences highlighted in red and in Fig. 3A, may represent the parts of interaction sites between αSyn and ClpP. As for other peptide inhibitors we developed [32–35], we coupled the ClpP and αSyn-derived peptides to the peptide carrier TAT_47−57_, which, we have shown, can quickly enter cells, pass through the blood-brain-barrier [32, 34, 35, 37, 43, 44], and achieve extensive bio-distribution within minutes in vivo and in culture [14, 33, 35, 37, 43–46].

We first determined whether CS1 or CS2 could block the interaction between αSyn and ClpP in vitro. While CS1 showed no effect, CS2 significantly reduced αSyn–ClpP binding (Fig. 3B). Consistent with this, CS2, but not CS1, abolished the binding of ClpP to WT-, ΔN-, and ΔC-αSyn (Fig. 3C–E; Fig. S2A), further supporting the selectivity of CS2 for disrupting the interaction. Streptavidin pull-down assays using biotin-labeled CS2 confirmed selective binding to αSyn but not to ClpP or LonP, another mitochondrial matrix protease (Fig. 3F). Microscale thermophoresis (MST) further revealed that CS2 directly bound αSyn monomer or PFF with a dissociation constant (Kd) of 7 or 4 µM, respectively, whereas the TAT control peptide exhibited no binding (Fig. 3G; Fig. S2B), confirming the specificity of the interaction. Functionally, CS2 restored ClpP peptidase activity in vitro following inhibition by either WT or A53T mutant αSyn (Fig. 3H, I). Notably, neither TAT nor CS2 alone impaired ClpP activity at concentrations up to 100 µM (Fig. S2C), indicating that CS2 is not intrinsically toxic and acts selectively by blocking αSyn-mediated inhibition.

Taken together, these findings identify CS2 as a rationally designed decoy peptide that selectively binds the NAC domain of αSyn and disrupts its pathological interaction with ClpP.

### CS2 treatment restores ClpP expression and attenuates αSyn-induced cytotoxicity

**Fig. 4:**
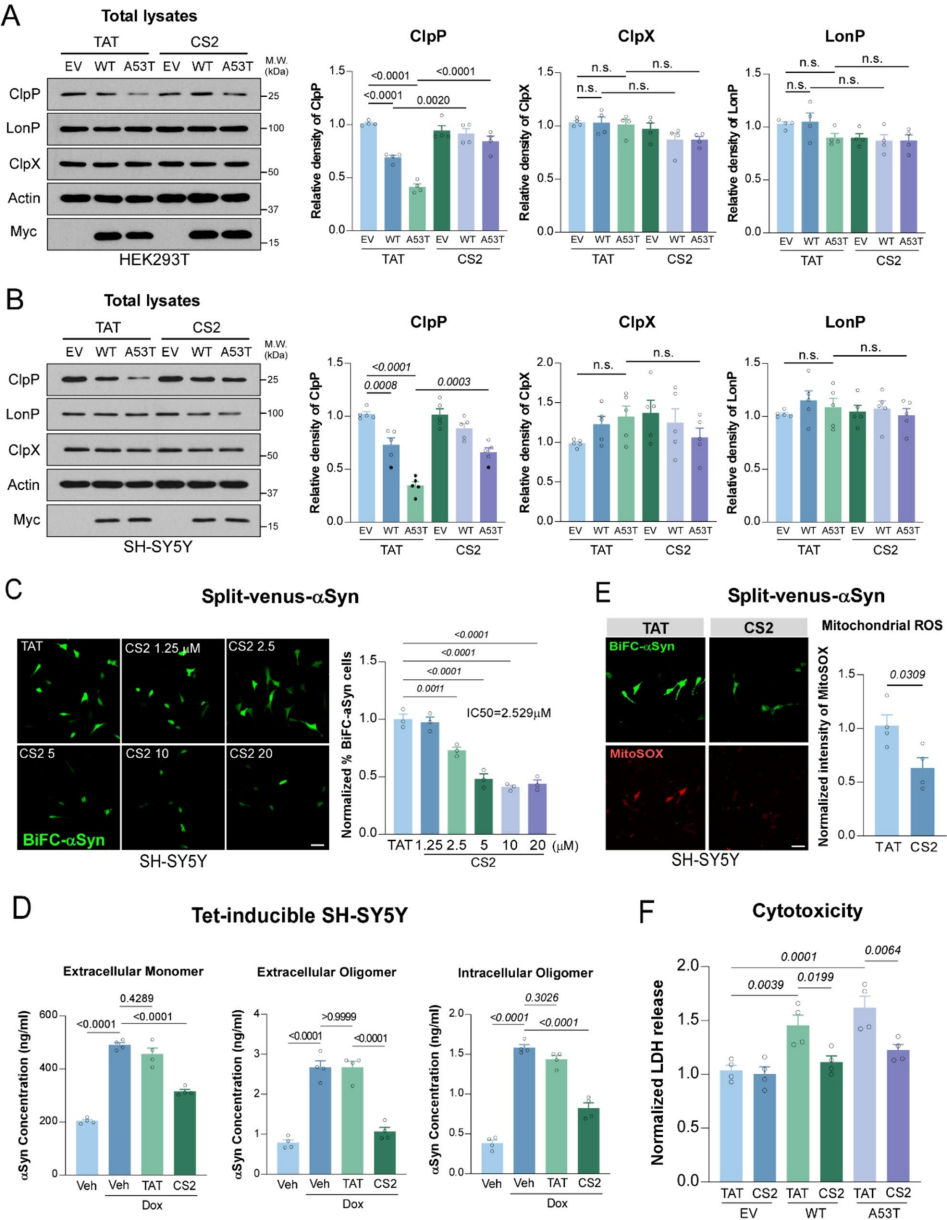
CS2 treatment rescues ClpP level and mitigates αSyn induced toxicity. (**A**-**B**) Western blot and quantification of ClpP, ClpX, and LonP in HEK293T or SH-SY5Y cells that were pre-treated with TAT or CS2 peptide 30 min before transfection of control vector (EV) or vectors expressing WT- or A53T-αSyn (*n* = 4–5, one-way ANOVA with Tukey’s *post-hoc* test; data are mean ± SEM). (**C**) Fluorescent images of split-Venus bimolecular αSyn in SH-SY5Y cells that were pre-treated with TAT or CS2 peptides at indicated doses before transfection of vectors expressing VN/VC-αSyn. The bimolecular fluorescence was examined 24-hour after transfection (*n* = 3, one-way ANOVA with Tukey’s *post-hoc* test, scale bar = 30 μm). (**D**) Quantification of the concentration (ng/ml) of extracellular αSyn monomer and oligomer, and intracellular αSyn oligomer in Tet-inducible SH-SY5Y cells that were pre-treated with TAT or CS2 peptide 30-minute before adding doxycycline (Dox) to induce αSyn expression. The concentration of αSyn species was determined by ELISA (*n* = 4, one-way ANOVA with Tukey’s *post-hoc* test; data are mean ± SEM). (**E**) Representative images of Mito-SOX staining in SH-SY5Y cells that were pre-treated with TAT or CS2 peptide and transfected with split Venus-αSyn vectors. Mitochondrial ROS production was determined by Mito-SOX staining after 24-hour transfection (*n* = 4, two-tailed Student’s t test, scale bar = 30 μm). (**F**) Quantification of the cytotoxicity in SH-SY5Y cells stably overexpressing EV, WT- or A53T-αSyn, which were treated with TAT or CS2 peptide for 2-day followed by 12-hour serum starvation. Cell death was determined by LDH assay (*n* = 4, one-way ANOVA with Tukey’s *post-hoc* test; data are mean ± SEM)

To confirm cellular uptake, we assessed the intracellular fluorescence of FITC-labeled CS2 in SH-SY5Y cells. CS2 rapidly entered cells, reaching peak intracellular levels within 30 min and remaining stable for at least 90 min after 1 µM treatment (Fig. S3A). Based on this uptake profile, all subsequent experiments employed a 30-minute pretreatment with CS2 or the TAT control peptide. In both SH-SY5Y and HEK293T cells, CS2 treatment significantly restored ClpP protein levels that had been suppressed by overexpression of WT or A53T αSyn (Fig. 4A, B). A similar effect was observed in Tet-inducible αSyn-expressing SH-SY5Y cells, where CS2 treatment led to a marked increase in ClpP levels compared to TAT-treated controls (Fig. S3B).

Because ClpP influences αSyn aggregation, we next examined whether CS2 could reduce αSyn oligomerization and associated cytotoxicity. In SH-SY5Y cells expressing VN/VC-αSyn, CS2 treatment significantly reduced BiFC signal intensity with an IC₅₀ of ~ 2.5 µM, indicating inhibition of αSyn oligomer formation (Fig. 4C). In Tet-inducible αSyn SH-SY5Y cells, CS2 treatment decreased levels of both extracellular and intracellular αSyn monomers and oligomers relative to TAT controls (Fig. 4D), suggesting that CS2 also limits αSyn release and spreading. Given that αSyn-induced ClpP loss caused mitochondrial oxidative stress [12], we then evaluated the effect of CS2 on the production of mitochondrial ROS. Comparing to TAT peptide, CS2 treatment resulted in significant reduction of the fluorescence intensity of Mito-SOX in SH-SY5Y cells expressing VN/VC-αSyn (Fig. 4E). Consistently, mitochondrial membrane potential and ATP content that are reduced in SH-SY5Y cells expressing αSyn were rescued upon CS2 treatment (Fig. S3C and D). However, the effects of CS2 on reducing αSyn oligomerization and mitochondrial ROS production were not observed in ClpP-deficient cells (Fig. S3E and F), indicating that the protective action of CS2 requires the presence of ClpP. Notably, CS2 did not affect cell viability nor ATP production in healthy SH-SY5Y cells (Fig. S3G and H), suggesting a low toxicity. In contrast, CS1, the peptide derived from homology sequence of αSyn (Fig. 3A), failed to restore ClpP expression (Fig. S4A and B), reduce αSyn oligomerization (Fig. S4C), or suppress ROS (Fig. S4D). These data support the specificity and selectivity of CS2. Furthermore, CS2 treatment significantly reduced cell death in SH-SY5Y cells expressing either WT- or A53T-αSyn (Fig. 4F). These findings demonstrate that CS2 depends on ClpP and attenuates αSyn-induced cytotoxicity.

### CS2 treatment alleviates αSyn-PFF induced cellular damage in primary neurons

**Fig. 5:**
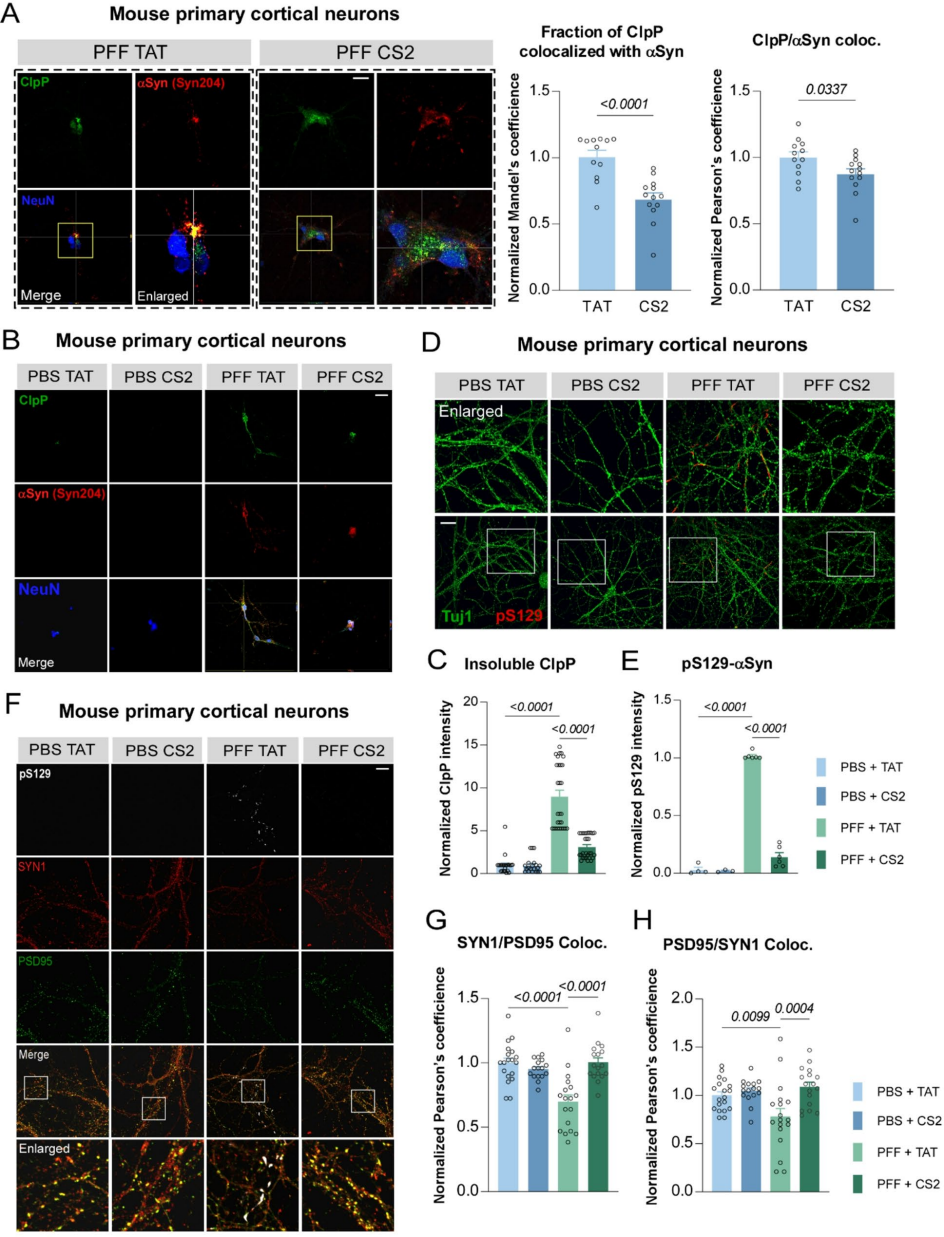
CS2 treatment mitigates αSyn PFF induced neurotoxicity. (**A**) Representative images of ClpP, αSyn (Syn204; recognize human αSyn only) and NeuN staining in αSyn PFF-inoculated mouse primary cortical neurons that were pre-treated with TAT or CS2 peptide (scale bar = 30 μm). Quantification of ClpP and αSyn co-localization (*n* = 12–13 neurons/group, two-tailed Student’s t test, data are mean ± SEM). (**B**-**C**) Representative images of ClpP, αSyn (Syn204) and NeuN staining in αSyn PFF-inoculated mouse primary cortical neurons that were pre-treated with TAT or CS2 peptide (scale bar = 30 μm). Neurons were fixed in 4% PFA/4% sucrose/1%Triton-X100 to extract the soluble factions before immunostaining. Quantification of fluorescence intensity of insoluble ClpP (*n* > 30 neurons/group, one-way ANOVA with Tukey’s *post-hoc* test; data are mean ± SEM). (**D**-**E**) Representative images of β-tubulin (Tuj1) and pS129-αSyn (pS129) staining in αSyn PFF-inoculated mouse primary cortical neurons that were pre-treated with TAT or CS2 peptide (scale bar = 30 μm). Quantification of fluorescence intensity of pS129 (*n* = 3–6, one-way ANOVA with Tukey’s *post-hoc* test; data are mean ± SEM). (**F**-**H**) Representative images of PSD95, Synapsin1 (SYN1) and pS129 staining in αSyn PFF-inoculated mouse primary cortical neurons that were pre-treated with TAT or CS2 peptide (scale bar = 30 μm). Quantification of PSD95 and SYN1 co-localization (*n* > 30 areas were quantitated; one-way ANOVA with Tukey’s *post-hoc* test; data are mean ± SEM)

We next evaluated the protective effect of CS2 in αSyn-PFF-inoculated primary cortical neurons, a well-established ex vivo model that recapitulates key aspects of αSyn-induced aggregation and neurotoxicity [47–49]. Primary neurons were treated with 1 µM CS2 or TAT control peptide daily for five consecutive days, starting at the time of αSyn-PFF inoculation (Fig. S5A). Consistent with earlier findings, ClpP was downregulated and co-localized with αSyn aggregates in PFF-inoculated neurons (Fig. 5A; Fig. S5B), but not in PBS-treated controls (Fig. S5C). Notably, CS2 treatment reduced this colocalization and rescued the expression of ClpP (Fig. 5A; Fig. S5B). Supporting this, we observed that ClpP, which typically localizes to the soluble mitochondrial fraction, became detectable in detergent-insoluble inclusions following PFF inoculation (Fig. 5B and C), indicative of co-aggregation with αSyn [50]. CS2 treatment significantly reduced the insoluble ClpP and αSyn signal (Fig. 5B and C; Fig. S5D), further demonstrating its ability to prevent pathological ClpP recruitment into αSyn aggregates. Additionally, CS2 treatment markedly reduced the level of pS129-αSyn, a pathological marker consistently observed in Lewy bodies of PD patient brains [51–53], in PFF-inoculated neurons (Fig. 5D and E; Fig. S5B), supporting that CS2 can mitigate αSyn aggregation and propagation.

In line with previous studies [49, 54], αSyn-PFF inoculation caused synaptic impairment, as evidenced by the uncoupling of pre- and post-synaptic proteins (Synapsin1 and PSD95) in the cortical neurons (Fig. 5F-H). Notably, CS2 treatment substantially restored Synapsin1/PSD95 colocalization (Fig. 5F–H), indicating preserved synaptic structure and function. Moreover, CS2 treatment reduced mitochondrial ROS and rescued mitochondrial membrane potential and ATP content in αSyn-PFF inoculated neurons (Fig. S5E-G). These findings demonstrate that CS2 protects against αSyn-PFF-induced neurotoxicity.

### CS2 treatment is protective in DA neurons derived from PD patient iPSCs

**Fig. 6:**
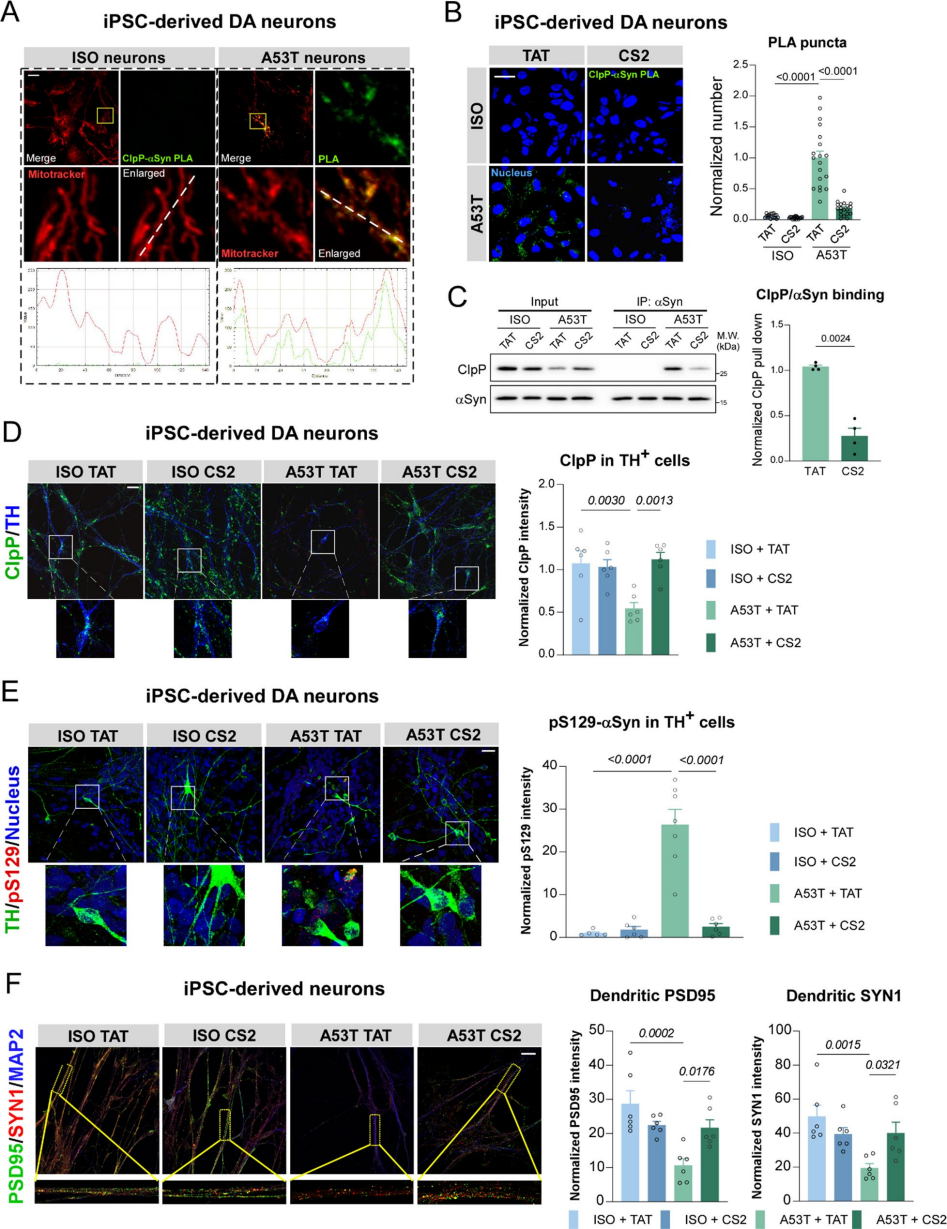
CS2 treatment is protective in DA neurons derived from PD patient iPSCs. (**A**) Representative images of MitoTracker staining and ClpP-αSyn PLA in A53T αSyn or isogenic control (ISO) iPSCs-derived neurons (scale bar = 30 μm). (**B**) Representative images of ClpP-αSyn PLA in A53T αSyn or ISO iPSCs-derived neurons, which were treated with TAT or CS2 peptide (scale bar = 30 μm). Quantification of the average PLA puncta number in neurons (*n* = 20 images per group, one-way ANOVA with Tukey’s *post-hoc* test; data are mean ± SEM). (**C**) Representative blots of co-immunoprecipitation assay assessing ClpP-αSyn in A53T αSyn or ISO iPSCs-derived neurons, which were treated with TAT or CS2 peptide. Normalized ratio of ClpP to αSyn was shown in histogram (*n* = 4, one-way ANOVA with Tukey’s *post-hoc* test; data are mean ± SEM). (**D**) Representative images of ClpP staining in tyrosine dehydrogenase positive (TH^+^) iPSCs-derived DA neurons treated with TAT or CS2 peptide (scale bar = 30 μm). Quantification of ClpP intensity in TH^+^ neurons (*n* = 6, one-way ANOVA with Tukey’s *post-hoc* test; data are mean ± SEM). (**E**) Representative images of pS129-αSyn (pS129) staining in TH^+^ iPSCs-derived DA neurons treated with TAT or CS2 peptide (scale bar = 30 μm). Quantification of pS129 intensity in TH^+^ neurons (*n* = 5–7, one-way ANOVA with Tukey’s *post-hoc* test; data are mean ± SEM). (**F**) Representative images of PSD95, Synapsin1 (SYN1) and MAP2 staining in iPSCs-derived neurons treated with TAT or CS2 peptide (scale bar = 30 μm). Quantification of the intensity of dendritic PSD95 and SYN1 (*n* = 6–7, one-way ANOVA with Tukey’s *post-hoc* test; data are mean ± SEM)

To further assess the neuroprotective effects of CS2 in a human-derived system, we used DA neurons differentiated from iPSCs of PD patients harboring the A53T mutation in αSyn. These patient-derived DA neurons exhibited ClpP downregulation, mitochondrial oxidative stress, and dendritic shortening [50], providing a clinically relevant platform for testing therapeutic interventions. Following our established protocol [14, 50], neurons differentiated from A53T-αSyn or isogenic control (ISO) iPSCs were treated with TAT or CS2 (1 µM) for five consecutive days (Fig. S6A). To determine whether the pathological interaction between endogenous ClpP and αSyn occurs within mitochondria, we performed in situ proximity ligation assay (PLA) combined with MitoTracker staining in neurons derived from human iPSCs. Only sparse ClpP–αSyn PLA puncta were detected in ISO neurons, whereas A53T-αSyn neurons exhibited increased PLA positive signals that strongly colocalized with MitoTracker-labeled mitochondria (Fig. 6A and B), consistent with the higher intramitochondrial levels of A53T-αSyn detected by mitochondrial fractionation (Fig. S6B). Co-immunoprecipitation further confirmed extensive ClpP–αSyn interaction in neurons derived from A53T-αSyn patient iPSCs (Fig. 6C), in agreement with our previous findings [50]. CS2 treatment markedly reduced the ClpP–αSyn interaction, as shown by both PLA and co-immunoprecipitation in A53T-αSyn neurons (Fig. 6B and C), and significantly restored ClpP protein levels in tyrosine hydroxylase (TH)-positive DA neurons (Fig. 6D). In parallel, CS2 reduced the expression of pS129-αSyn and the accumulation of insoluble αSyn, both of which were markedly elevated in A53T-αSyn neurons compared to ISO-derived neurons (Fig. 6E; Fig. S6C), consistent with inhibition of pathological αSyn aggregation. As previously reported [55], A53T-αSyn patient-derived neurons exhibited pronounced dendritic loss and synaptic impairments (Fig. 6F); notably, CS2 treatment restored the density of both PSD95 and Synapsin1 in these neurons (Fig. 6F). Moreover, CS2 reduced mitochondrial ROS and rescued mitochondrial membrane potential and ATP content in neurons derived from A53T-αSyn iPSCs (Fig. S6D–F). Together, these results demonstrate that CS2 counteracts αSyn-induced ClpP deficiency, reduces pathological αSyn accumulation, and restores neuronal, synaptic, and mitochondrial integrity in patient-derived DA neurons, supporting its potential as a disease-modifying therapeutic agent for PD.

### CS2 treatment alleviates αSyn-associated neuropathology and behavioral deficits in vivo

Lastly, to evaluate the therapeutic potential of CS2 in vivo, we tested its efficacy in the mThy1-hSNCA transgenic mouse model (Line 15), which overexpresses wild-type human αSyn under the Thy1 promoter. This mouse model exhibits progressive αSyn accumulation, neuroinflammation, and motor and cognitive deficits [56–58]. In addition to the late stage (age of 8―10 months) PD-associated neuropathology and motor deficits, mThy1-hSNCA mice exhibit early-stage (age of 4.5―6 months) cognitive impairments reminiscent of dementia with Lewy Bodies (DLB) [59]. We first conducted a dose-ranging pilot study in mThy1-hSNCA mice beginning at 4 months of age, a stage characterized by early cognitive deficits. Mice received subcutaneous CS2 (1, 3, or 10 mg/kg/day) or TAT control peptide for 8 weeks via osmotic mini pumps (Fig. S7A). In the Y-maze test at 6 months, CS2 treatment improved spontaneous alternation behavior in a dose-dependent manner, with no significant difference between 3 and 10 mg/kg/day (Fig. S7B). At both doses, CS2 restored ClpP expression in the midbrain and reduced levels of soluble pS129-αSyn and detergent-insoluble αSyn (Fig. S7C, D). Moreover, CS2 treatment at 3 mg/kg/day can abolish the interaction between ClpP and αSyn in mThy1-hSNCA mouse midbrain, but without disparity from 10 mg/kg/day (Fig. S7E). These findings indicate that 3 mg/kg/day achieves significant efficacy with minimal dosing and were thus selected for subsequent experiments.

**Fig. 7:**
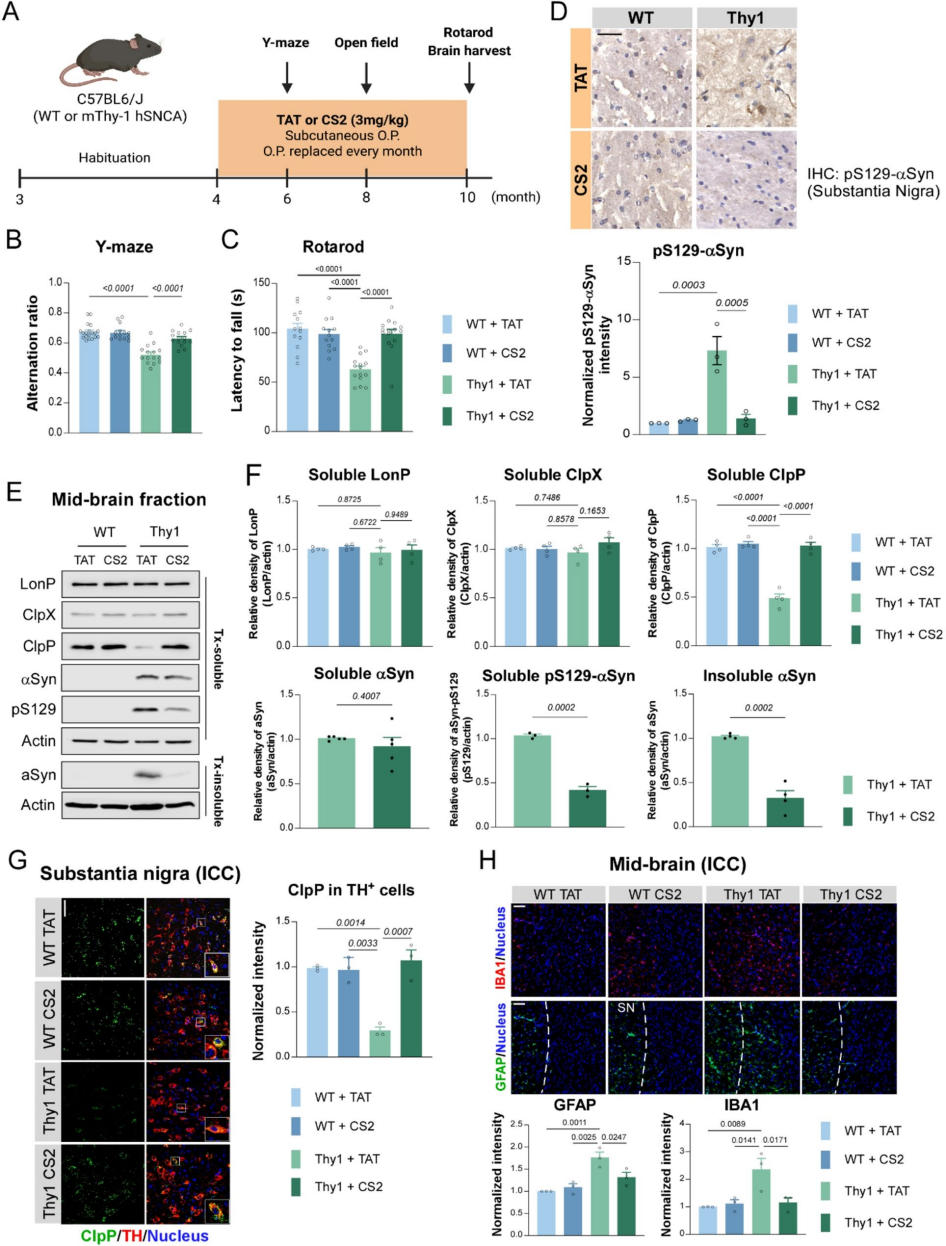
CS2 treatment is protective in mThy1-hSNCA mouse model of PD. (**A**) Experimental schematic of CS2 treatment in mThy1-hSNCA (Thy1) mouse model of PD: TAT or CS2 peptide was administered (3 mg/kg/day) via subcutaneous osmotic pump (O.P.) in Thy1 mice and their wild-type (WT) littermates starting from age of 4-month till 10-month. (**B**) Quantification of alternation ratio evaluated by Y-maze at age of 6-month (*n* = 15 mice/group, one-way ANOVA with Tukey’s *post-hoc* test; data are mean ± SEM). (**C**) Quantification of latency to fall (second; s) in Rotarod acceleration test conducted at age of 10-month (*n* = 13–15 mice/group, one-way ANOVA with Tukey’s *post-hoc* test; data are mean ± SEM). (**D**) Representative images of pS129-αSyn staining in substantia nigra of WT and Thy1 mice treated with TAT or CS2 peptide (scale bar = 100 μm). Quantification of pS129-αSyn intensity (*n* = 3 mice/group, one-way ANOVA with Tukey’s *post-hoc* test; data are mean ± SEM). (**E**-**F**) Western blot and quantification of LonP, ClpX, ClpP, αSyn and pS129-αSyn in midbrain soluble fraction, and αSyn in midbrain insoluble fraction, which were harvested from 10-month-old WT or Thy1 mice (*n* = 3–5 mice/group, one-way ANOVA with Tukey’s *post-hoc* test; data are mean ± SEM). (**G**) Representative images of ClpP and tyrosine dehydrogenase (TH) staining on the brain coronal sections from TAT or CS2-treated Thy1 mice and their WT littermates (scale bar = 100 μm). Quantification of ClpP intensity in TH^+^ neurons (*n* = 3 mice/group, one-way ANOVA with Tukey’s *post-hoc* test; data are mean ± SEM). (**H**) Representative images of IBA1 and GFAP staining in the midbrain of TAT or CS2 treated Thy1 mice and their WT littermates (SN: substantia nigra) (scale bar = 180 μm). Quantification of the fluorescence intensity (per 4.2 mm^2^) of IBA1 and GFAP (*n* = 3 mice/group, one-way ANOVA with Tukey’s *post-hoc* test; data are mean ± SEM)

Sustained subcutaneous CS2 administration at 3 mg/kg/day (Fig. 7A) significantly improved both cognitive and motor performance in mThy1-hSNCA mice. CS2-treated mThy1-hSNCA mice showed improved cognitive activity in the Y-maze (Fig. 7B), enhanced motor coordination on the rotarod (Fig. 7C) and ameliorated locomotor activity in the open-field test (Fig. S8A), compared to TAT-treated diseased mice. Notably, CS2 had no observable effects on wild-type littermates, supporting its safety.

Neuropathological analysis revealed significant accumulation of pS129-αSyn and αSyn aggregates in the SN of mThy1-hSNCA mice, which was reduced by CS2 treatment (Fig. 7D; Fig. S8B), confirming its ability to suppress αSyn aggregation in vivo. Immunohistochemistry and Western blotting showed that ClpP protein levels were selectively decreased in midbrain DA neurons of mThy1-hSNCA mice, a deficit that was restored by CS2 without affecting other mitochondrial matrix proteins (Fig. 7E–G). Such ClpP downregulation is likely attributed to the accumulation of intramitochondrial αSyn (Fig. S8C), which promotes the redistribution of ClpP from soluble to insoluble fractions (Fig. S8D), in line with our previous findings [50]. Given the contribution of neuroinflammation to PD pathogenesis [60, 61], we next evaluated microglial and astrocytic activation. The mThy1-hSNCA mice exhibited elevated IBA1 (a marker of microglia) and GFAP (a marker of astrocyte) fluorescence intensity in the midbrain, indicative of reactive gliosis. CS2 treatment significantly attenuated both markers, with no effect in WT mice (Fig. 7H). Taken together, these findings indicate that CS2 treatment attenuates αSyn-associated neuropathology and neuroinflammation in vivo by disrupting ClpP-αSyn interaction.

**Fig. 8:**
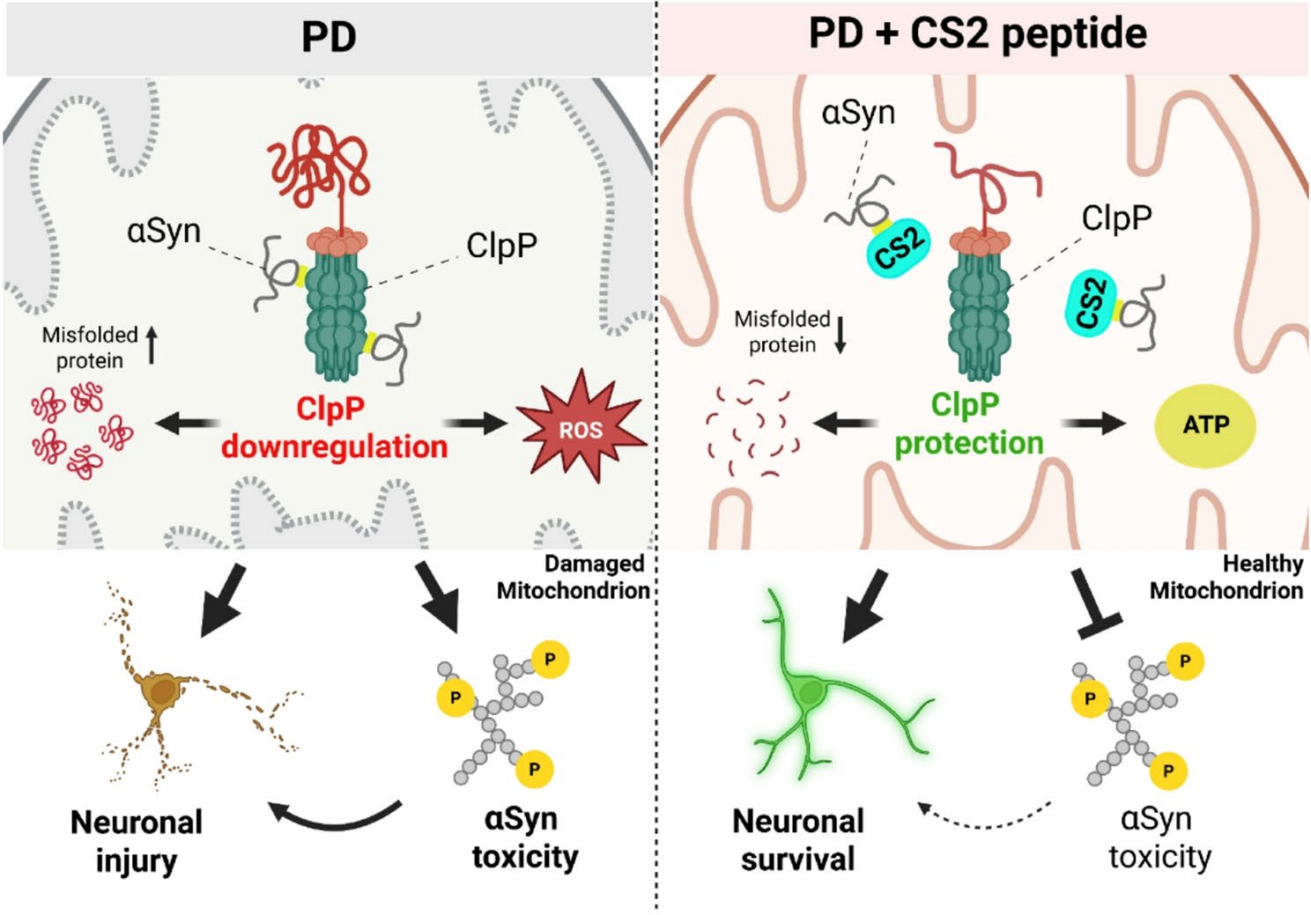
Graphic summary of the findings

## Discussion

This study uncovers a pathological mechanism in PD involving the direct interaction between αSyn and the mitochondrial protease ClpP (Fig. 8). We demonstrate that ClpP negatively regulates αSyn aggregation and propagation, likely by stabilizing native αSyn tetramers. Mechanistically, we identify the NAC domain of αSyn as the interface for ClpP binding and show that this interaction suppresses ClpP level and protease activity. Disrupting this interaction with a rationally designed decoy peptide-CS2-restores ClpP function and attenuates αSyn-induced neurotoxicity across multiple models. CS2 not only reduces αSyn aggregation in vitro and in neurons derived from PD patient iPSCs but also mitigates neuropathology, neuroinflammation, and motor and cognitive deficits in αSyn transgenic mice. Together, these findings indicate the αSyn–ClpP interaction as a targetable driver of PD pathogenesis and provide preclinical evidence for CS2 as a potential therapeutic agent.

Emerging evidence suggests that αSyn naturally exists as a helically folded tetramer, which is resistant to aggregation [16]. Disrupting αSyn tetramerization leads to the accumulation of unfolded monomers, facilitating and accelerating αSyn aggregation and propagation in PD pathogenesis [16]. Thus, αSyn tetramerization plays a critical role in controlling the formation of toxic αSyn species, and understanding its regulation may provide important insights into the etiology of PD. Our findings reveal that ClpP plays a previously unrecognized role in supporting αSyn tetramer stability; ClpP deficiency is sufficient to shift αSyn from its tetrameric to monomeric form, whereas ClpP overexpression increased the αSyn tetramer-to-monomer ratio. Notably, overexpression of a proteolytically inactive ClpP-S153A mutant significantly reduced αSyn tetramer levels. Since ClpP-S153A overexpression leads to the accumulation of potential proteolytic substrates such as ERAL1 [62] and NDUFS1 [63], it is plausible that these or other ClpP substrates, along with their downstream effectors, may also influence the αSyn tetramer pool. Therefore, future studies aimed at identifying neuron-specific ClpP substrates may elucidate the detailed mechanisms by which ClpP modulates αSyn tetramerization and aggregation, potentially revealing novel regulatory pathways and therapeutic targets for PD.

Several lines of evidence support that a fraction of αSyn is actively transported into mitochondria and can access intramitochondrial compartments, including the matrix. Early import studies in DA neurons identified a cryptic N-terminal mitochondrial targeting sequence in αSyn and showed that its mitochondrial entry is membrane-potential dependent and requires the TOM40 import pore [5], consistent with translocation across both mitochondrial membranes. Biochemical fractionation and imaging analyses have detected αSyn at the inner mitochondrial membrane and within the matrix [64, 65], although the exact stoichiometry and import routes remain debated. Notably, accumulation of endogenous αSyn inside DA neuron mitochondria appears to increase with physiological aging and pathological stress [50, 66]. Together, these findings provide key mechanistic support for our observation that αSyn interacts with and suppresses the mitochondrial matrix protease ClpP in PD models. Because ClpP resides in the matrix, a direct physical interaction requires αSyn to cross at least the outer membrane and approach the matrix side of the inner membrane. The demonstration that αSyn contains a cryptic mitochondrial targeting sequence, engages canonical protein-import machinery, and is detectable in IMM/matrix fractions makes it plausible that a subset of αSyn reaches the same compartment as ClpP and directly perturbs intramitochondrial proteostasis, consistent with our data that αSyn–ClpP interaction drives oxidative stress and neurotoxicity in PD-relevant neurons.

Current treatments for PD, including levodopa-like agents, dopamine agonists, and surgical interventions such as deep brain stimulation, primarily alleviate motor symptoms without altering disease progression [67, 68]. Over time, these therapies often become less effective and are associated with significant side effects, such as dyskinesia. Moreover, effective treatments for the debilitating non-motor symptoms of PD remain limited, and no existing therapy can halt or slow the underlying neurodegenerative process. Given that αSyn aggregation and mitochondrial dysfunction are central pathological features of PD, therapeutics that disrupt the vicious cycle between these two processes may offer promising disease-modifying strategies. Our findings demonstrate that CS2 treatment confers neuroprotection in models of αSyn-induced toxicity, providing proof of concept that peptide inhibitors targeting the mitochondria–αSyn interaction may represent a novel therapeutic approach for PD. We have previously developed several decoy peptides, such as P110 [33] and DA1 [34] to preserve mitochondrial function by selectively inhibiting key protein interactions. These peptides may also hold therapeutic potential for other neurodegenerative or neurological disorders that share mitochondrial dysfunction as a central pathological mechanism. Similarly, the αSyn–ClpP interaction may be a relevant pathological event in other αSynopathies beyond PD. If so, CS2 peptides may benefit many αSyn-related disorders.

Our results demonstrate the high specificity and selectivity of CS2. Notably, the CS2 peptide is derived from a region of ClpP that aligns with the NAC domain of αSyn, whereas the CS1 peptide is derived from αSyn. Although the CS1 and CS2 sequences exhibit high similarity, CS1 showed no effect, either in vitro or in vivo, on αSyn-ClpP interaction or αSyn aggregation, highlighting the selective activity of CS2. Importantly, while the proposed CS2 binding pocket contains several key residues critical for forming the unfolded core of αSyn fibrils, we observed no protective effect of CS2 on αSyn aggregation or mitochondrial function in ClpP-deficient cells. This indicates that CS2 requires the presence of ClpP and specifically targets the pathological ClpP–αSyn interaction, rather than acting as a general inhibitor of αSyn aggregation. Consistent with this, CS2 binds both monomeric and fibrillar αSyn in vitro, yet exhibits protective activity only when ClpP is present. Because mitochondrial import mechanisms favor monomeric αSyn [69, 70], we propose a mechanistic model in which monomeric αSyn is transported into mitochondria, engages the matrix protease ClpP, and undergoes pathological interaction that can be selectively disrupted by CS2.

## Conclusion

In summary, our study identifies the αSyn–ClpP interaction as a critical pathological link between mitochondrial dysfunction and αSyn aggregation in PD. Targeting this interaction with the decoy peptide CS2 restores mitochondrial proteostasis and attenuates αSyn-induced toxicity, offering a promising therapeutic strategy for PD and other synucleinopathies. Future studies leveraging additional models, such as αSyn PFF-inoculated mice and patient-derived brain organoids, will be important to further evaluate the neuroprotective effects of CS2 on nigrostriatal degeneration. In parallel, comprehensive pharmacokinetic and pharmacodynamic profiling will support the translational potential of CS2 for clinical applications.

## Supplementary Information

Below is the link to the electronic supplementary material.

Supplementary Material 1

Supplementary Material 2

## Data Availability

All data are available in the main text or the supplementary materials. All data generated and analyzed during this study are included in this article or available from the corresponding author on reasonable request.

## Abbreviations

PD: Parkinson’s disease
DA: Dopaminergic
SN: Substantia Nigra
αSyn: α-Synuclein
ROS: Reactive oxygen species
iPSCs: Induced pluripotent stem cells
WT: Wild-type
NAC: Non-amyloid β component
ΔN: Lacking N-terminal domain
ΔC: Lacking C-terminal domain
ΔNAC: Lacking NAC domain
PFF: Preformed fibril
BiFC: Bimolecular fluorescence complement
ThT: Thioflavin T
Co-IP: Co-immunoprecipitation
MST: Microscale thermopherosis
PLA: Proximity ligation assay
