# Supplementary material for: Disrupting α-Synuclein–ClpP interaction restores mitochondrial function and attenuates neuropathology in Parkinson’s disease models
Source: Mol Neurodegener. 2025 Dec 22;20:126. doi: 10.1186/s13024-025-00918-w (PMC12751141; doi:10.1186/s13024-025-00918-w)
Supplement: Supplementary file 1 — Supplementary Material 1 [file 13024_2025_918_MOESM1_ESM.docx]

**Supplementary Information**

**Disrupting αSynuclein–ClpP interaction restores mitochondrial function and attenuates neuropathology in Parkinson’s Disease models**

Di Hu^1,2^, Xiaoyan Sun^1^, Xin Qi^1,2*^

^1^ Department of Physiology & Biophysics, Case Western Reserve University School of Medicine, Cleveland, OH 44106, USA; ^2^ Center for Mitochondrial Research and Therapeutics, Case Western Reserve University School of Medicine, Cleveland, OH 44106, USA

*Corresponding author: Xin Qi PhD, Email: [xxq38@case.edu](mailto:xxq38@case.edu). Department of Physiology and Biophysics, Case Western Reserve University School of Medicine, 10900 Euclid Ave, E516, Cleveland, OH 44106-4970, USA

**
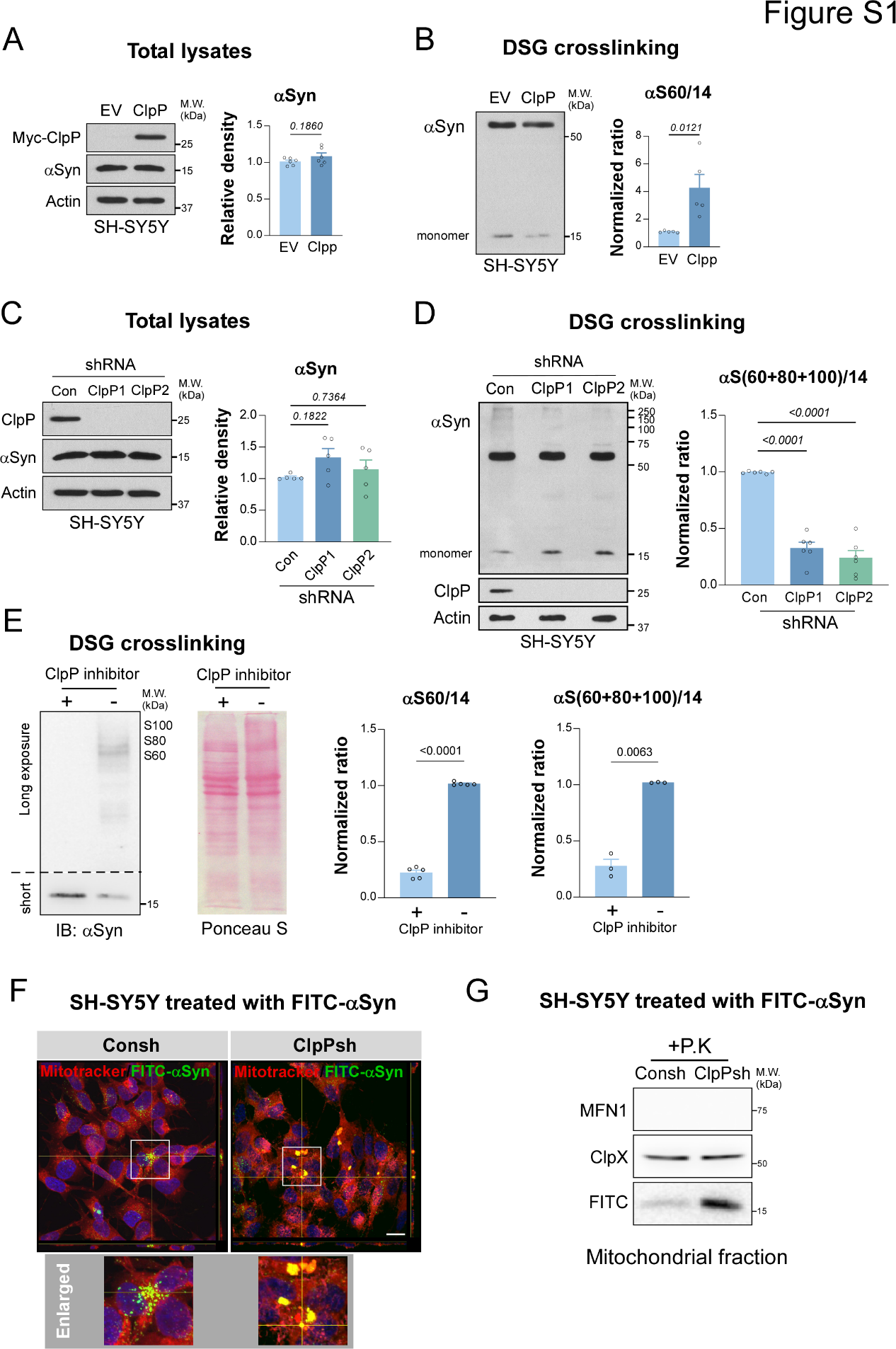
**

**Figure S1. ClpP modulates αSyn tetramers.**

**(A)** Western blot and quantification of endogenous αSyn in SH-SY5Y cells transfected with control vector (EV) or Myc-tagged ClpP vector (n = 6, two-tailed Student’s t test, data are mean ± SEM).

**(B)** Western blot and quantification of endogenous αSyn tetramer in SH-SY5Y cells overexpressing EV or Myc-ClpP upon intact cell crosslinking by DSG (n = 5, two-tailed Student’s t test, data are mean ± SEM).

**(C)** Western blot and quantification of endogenous αSyn in control knock-down (con shRNA) or ClpP knock-down (ClpP 1 or 2 shRNA) SH-SY5Y cells (n = 5, two-tailed Student’s t test, data are mean ± SEM).

**(D)** Western blot and quantification of endogenous αSyn oligomer in Con shRNA or ClpP shRNA SH-SY5Y cells upon intact cell crosslinking by DSG (n = 6, two-tailed Student’s t test, data are mean ± SEM).

**(E)** Western blot and quantification of endogenous αSyn oligomer in control iPSC-derived neurons treated with ClpP inhibitor or not (n = 5, two-tailed Student’s t test, data are mean ± SEM).

**(F)** Representative images of Mito-tracker staining in Consh or ClpPsh SH-SY5Y cells treated with FITC- αSyn (scale bar=20 µm).

**(G)** Mitochondrial fractions were isolated from the consh and ClpPsh expressing SH-SY5Y cells treated with FITC-αSyn, and subjected for western blot after proteinase-K digestion. Shown are the representative blots of 3 independent experiments.

**
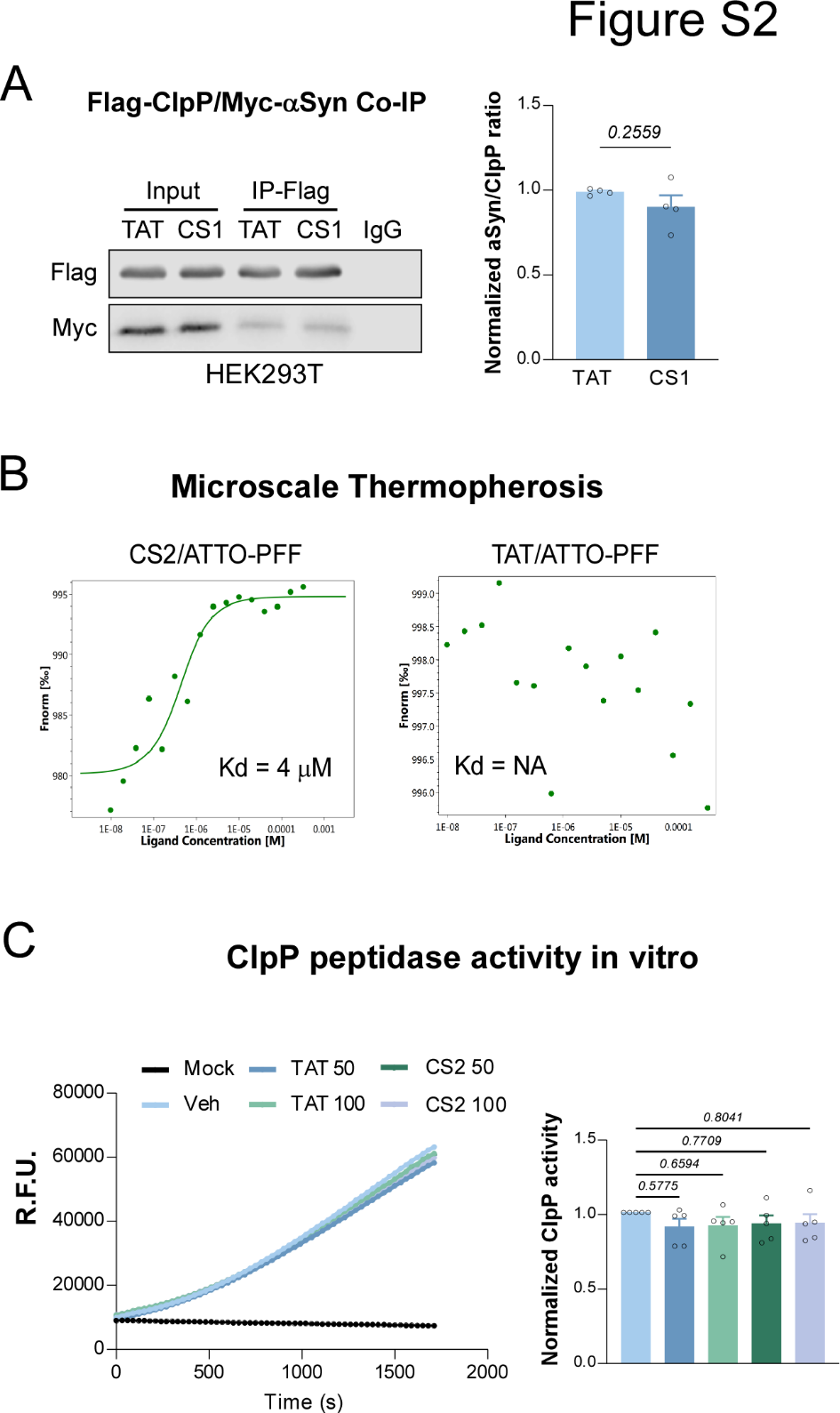
**

**Figure S2. Development of CS1/CS2 decoy peptides.**

**(A)** Total lysates were harvested from TAT or CS1-treated HEK293T cells overexpressing Flag-tagged ClpP with Myc-tagged αSyn, and subjected for immunoprecipitation using anti-Flag antibody, followed by western blot analyses. Shown are representative blots of 3 independent experiments. Quantification of the ratio of αSyn over ClpP (pull-down) (n = 4, two-tailed Student’s t test, data are mean ± SEM).

**(B)** The binding affinity between ATTO-αSyn-PFF and CS2 or TAT was measured by microscale thermophoresis. Shown are the representative repeat of 4 independent experiments. Mean Kd value is shown.

**(C)** *In vitro* ClpP peptidase activity was measured in presence of TAT or CS2 peptide (50/100 µM). The fluorescence intensity of ac-WLA-AMC (50 µM), a fluorogenic substrate of ClpP, was measured (0-30 min). Quantification of ClpP activity (n = 5, one-way ANOVA with Tukey’s *post-hoc* test; data are mean ± SEM).

**
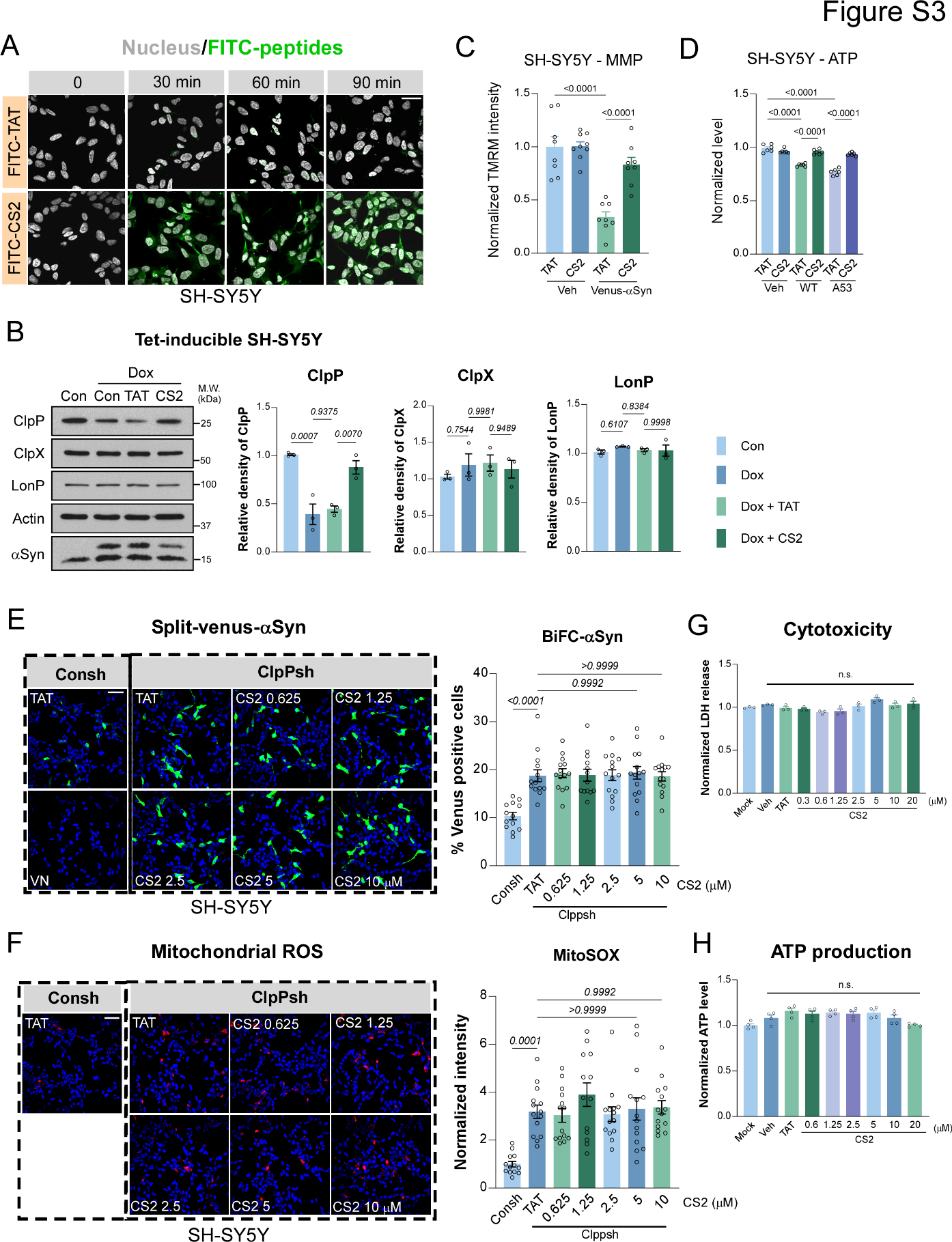
**

**Figure S3. CS2 targets ClpP-αSyn interaction.**

**(A)** Representative images of SH-SY5Y cells that were treated with FITC-conjugated TAT or CS2 peptide (10 μM). Cells were fixed at the indicated time points for confocal imaging (scale bar= 30µm).

**(B)** Western blot and quantification of ClpP, ClpX and LonP in Tet-inducible SH-SY5Y cells that were treated with TAT or CS2 peptide (n = 3, one-way ANOVA with Tukey’s *post-hoc* test; data are mean ± SEM).

**(C)** Quantification of TMRM staining in SH-SY5Y cells expressing Venus-αSyn or control vector (Veh) after treatment with TAT or CS2 peptide (n = 8, one-way ANOVA with Tukey’s *post-hoc* test; data are mean ± SEM).

**(D)** Quantification of ATP level in SH-SY5Y cells expressing WT- or A53T- αSyn or control vector (Veh) after treatment with TAT or CS2 peptide (n = 6, one-way ANOVA with Tukey’s *post-hoc* test; data are mean ± SEM).

**(E)** Representative images of split-Venus bimolecular αSyn in control knock-down (Consh) or ClpP knock-down (ClpPsh) SH-SY5Y cells that were pre-treated with TAT or CS2 peptides at indicated doses before transfection of VN/VC-αSyn-expressing vectors (scale bar = 30 µm). The bimolecular fluorescence was examined 24-hour after the transfection and quantitated (n = 14 areas/group, one-way ANOVA with Tukey’s *post-hoc* test, data are mean ± SEM).

**(F)** Representative images of Mito-SOX staining in Consh or ClpPsh SH-SY5Y cells overexpressing αSyn, which were pre-treated with TAT or CS2 peptide (scale bar = 30 µm). Quantification of the fluorescence intensity of Mito-SOX (n = 13 areas/group, one-way ANOVA with Tukey’s *post-hoc* test, data are mean ± SEM).

**(G)** Quantification of cytotoxicity examined by LDH assay in SH-SY5Y cells treated with indicated peptides (n = 3, one-way ANOVA with Tukey’s *post-hoc* test, data are mean ± SEM).

**(H)** Quantification of ATP level in SH-SY5Y cells treated with indicated peptides (n = 4, one-way ANOVA with Tukey’s *post-hoc* test, data are mean ± SEM).

**
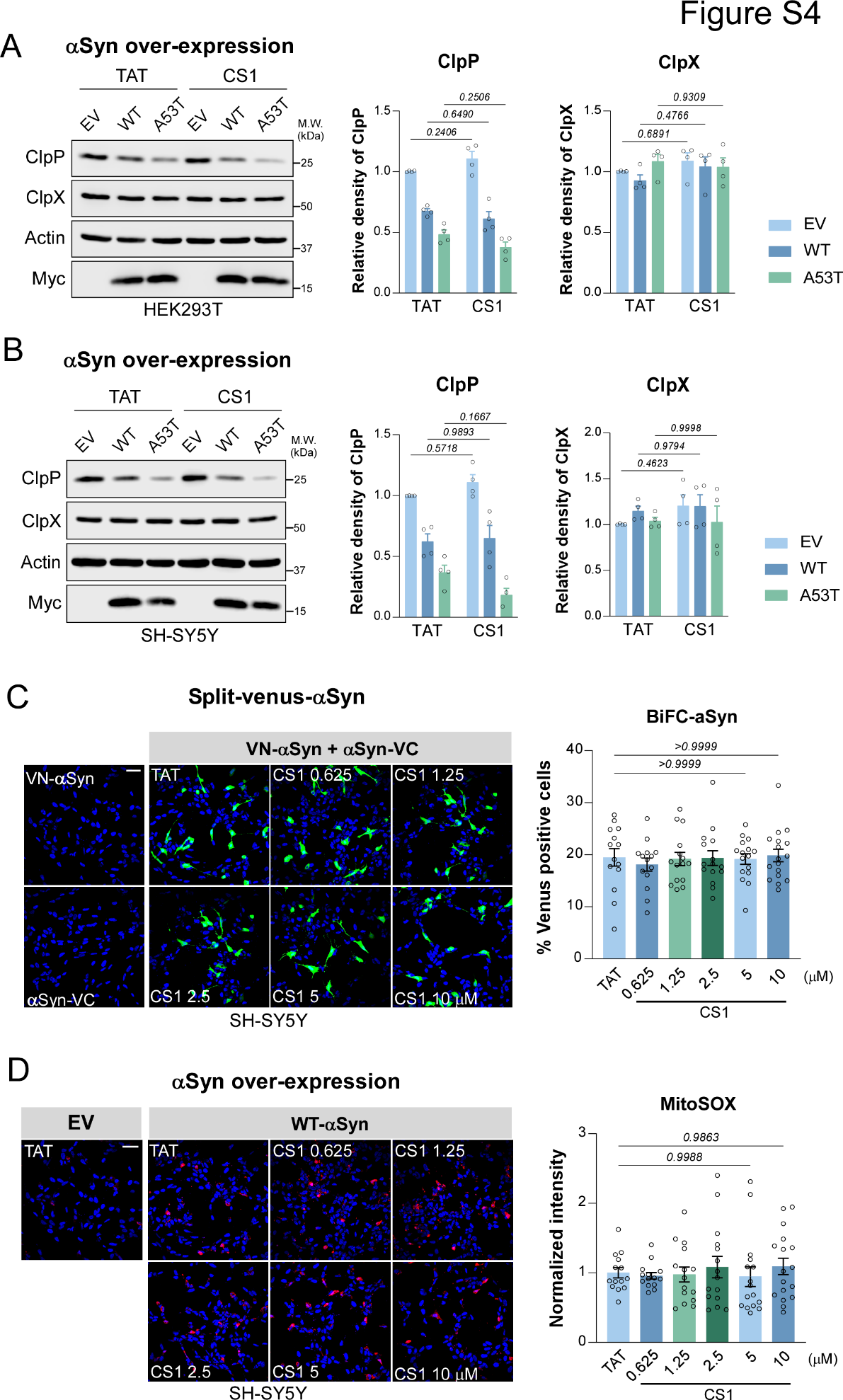
**

**Figure S4. CS1 has no effect in cell culture.**

**(A-B)** Western blot and quantification of ClpP and ClpX in TAT- or CS1-treated HEK293T and SH-SY5Y cells over-expressing wild-type (WT) or mutant A53T αSyn (n = 4, one-way ANOVA with Tukey’s *post-hoc* test, data are mean ± SEM).

**(C)** Representative images of split-Venus bimolecular αSyn in SH-SY5Y cells that were pre-treated with TAT or CS2 peptides at indicated doses before transfection of VN- or/and VC-αSyn -expressing vectors (scale bar = 30 µm). The bimolecular fluorescence was examined 24-hour after the transfection and quantitated (n = 14 areas/group, one-way ANOVA with Tukey’s *post-hoc* test, data are mean ± SEM).

**(D)** Representative images of Mito-SOX staining in SH-SY5Y cells overexpressing wild-type (WT) αSyn, which were pre-treated with TAT or CS2 peptide (scale bar = 30 µm). Quantification of the fluorescence intensity of Mito-SOX (n = 14 areas/group, one-way ANOVA with Tukey’s *post-hoc* test, data are mean ± SEM).

**
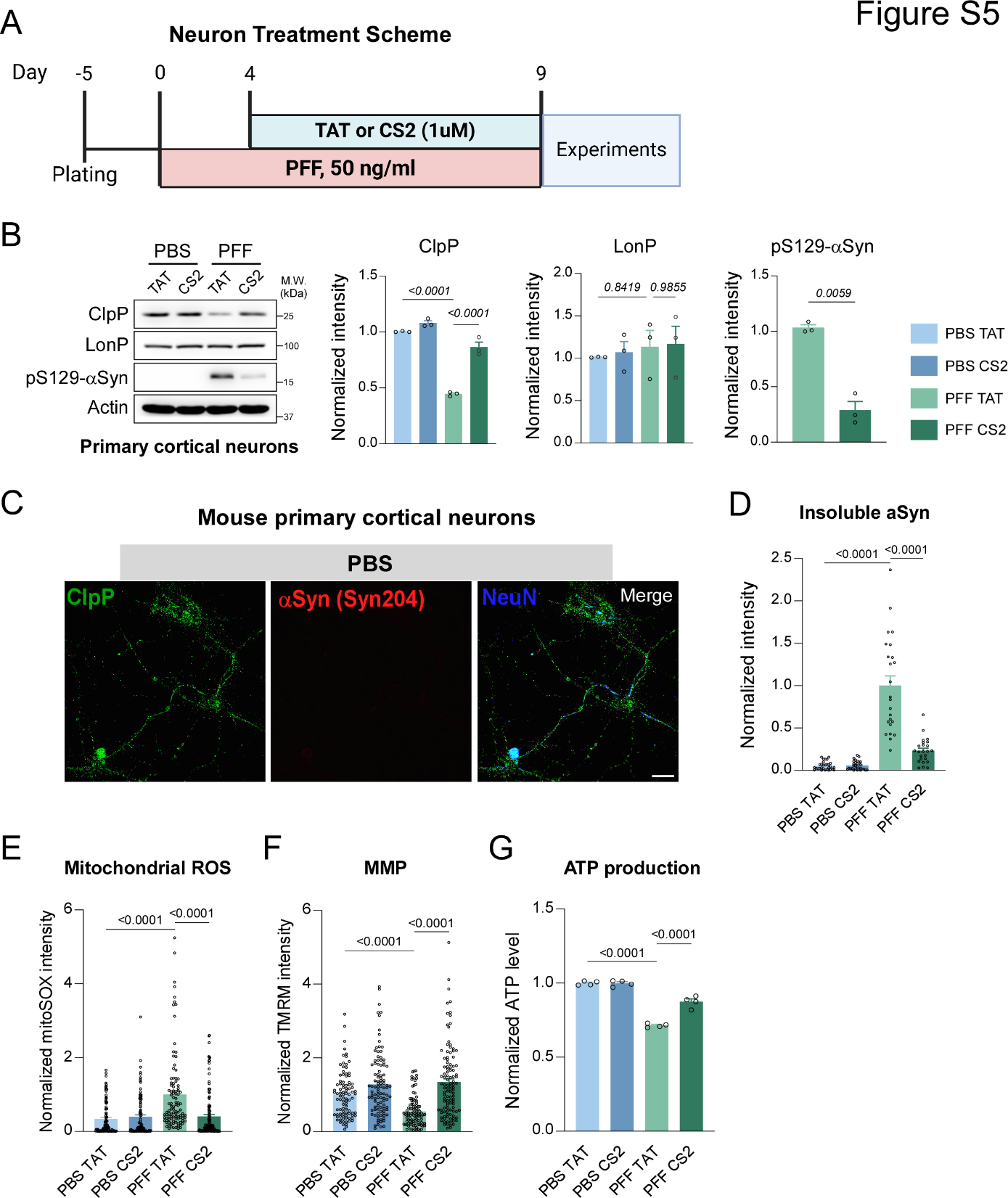
**

**Figure S5. CS2 treatment is protective in αSyn PFF inoculated primary cortical neurons.**

**(A)** Experimental schematic of TAT or CS2 peptide treatment in mouse primary cortical neurons inoculated with human αSyn PFF: Primary cortical neurons were isolated from E18 mouse cortex (see methods) and plated on coverslips (Day -5); neurons were treated with human αSyn PFF on Day 0 at 50 ng/ml; On Day 4, TAT or CS2 (1µM) was added daily till Day 9.

**(B)** Total protein lysates were harvested from primary neurons inoculated with αSyn-PFF after treatment with TAT or PBS, and subjected for western blot. Shown are the representative blots. Histogram summarize the results of 3 independent experiments (one-way ANOVA with Tukey’s *post-hoc* test, data are mean ± SEM).

**(C)** Representative images of ClpP, αSyn (Syn204) and NeuN in primary cortical neurons treated with PBS. Syn204 antibody specifically recognizes human αSyn, thus no signal was detected in PBS-treated neurons.

**(D)** Quantification of fluorescence intensity of αSyn in the insoluble fraction of the primary neurons after indicated treatment (n = 20-30 neurons per group, one-way ANOVA with Tukey’s *post-hoc* test, data are mean ± SEM).

**(E)** Quantification of Mito-SOX staining in the primary neurons (n= 90-100 neurons per group, one-way ANOVA with Tukey’s *post-hoc* test, data are mean ± SEM).

**(F)** Quantification of TMRM staining in the primary neurons (n= 90-100 neurons per group, one-way ANOVA with Tukey’s *post-hoc* test, data are mean ± SEM).

**(G)** Shown are the normalized ATP level in the primary neurons with indicated treatment (n= 4 independent repeats, one-way ANOVA with Tukey’s *post-hoc* test, data are mean ± SEM).

**
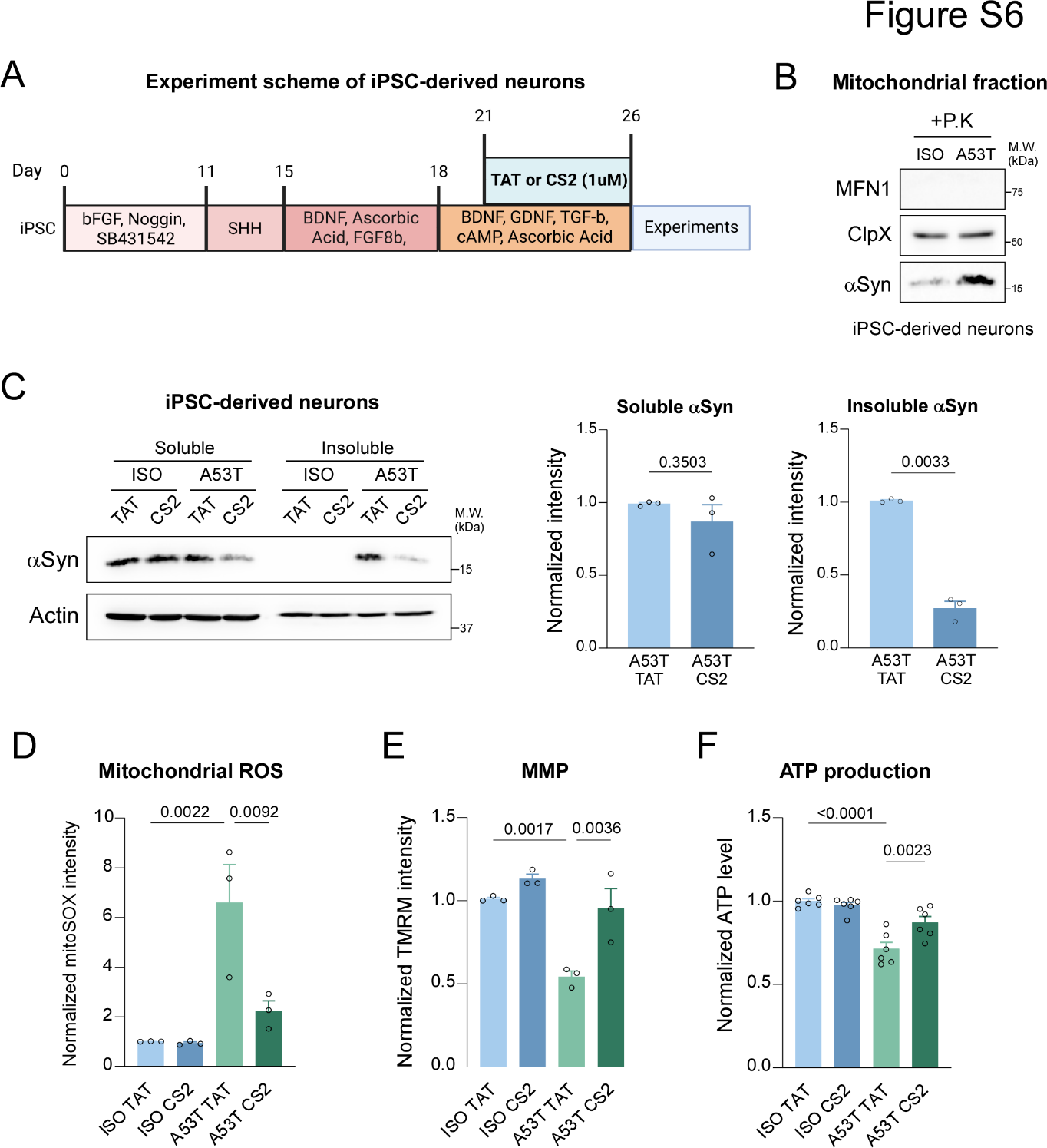
**

**Figure S6. CS2 treatment is protective in PD patient’s iPSC-derived neurons.**

**(A)** Experimental schematic of CS2 treatment in iPSCs-derived neurons: A53T-αSyn iPSCs (A53T) and its isogenic control iPSCs (ISO) were differentiated into dopaminergic (DA) neurons for 26 days. Neurons were treated with TAT or CS2 peptide (1μM) for 5 consecutive days starting from Day 21, and then fixed for the following experiments.

**(B)** Mitochondrial fractions were isolated from the ISO and A53T iPSC-derived neurons, and subjected for western blot after proteinase-K digestion. Shown are the representative blots of 3 independent experiments (one-way ANOVA with Tukey’s *post-hoc* test, data are mean ± SEM).

**(C)** Detergent soluble and insoluble fractions were extracted from iPSC-derived neurons and subjected for western blot analyses. Shown are the representative blots of 3 independent experiments. Histogram shows normalized intensity of αSyn in the soluble or insoluble fractions (one-way ANOVA with Tukey’s *post-hoc* test, data are mean ± SEM).

**(D)** Quantification of Mito-SOX staining in the iPSC-derived neurons (n= 3 independent experiments, one-way ANOVA with Tukey’s *post-hoc* test, data are mean ± SEM).

**(E)** Quantification of TMRM staining in the iPSC-derived neurons (n= 3 independent experiments, one-way ANOVA with Tukey’s *post-hoc* test, data are mean ± SEM).

**(F)** Shown are the normalized ATP level in the primary neurons with indicated treatment (n= 6 independent experiments, one-way ANOVA with Tukey’s *post-hoc* test, data are mean ± SEM).

**
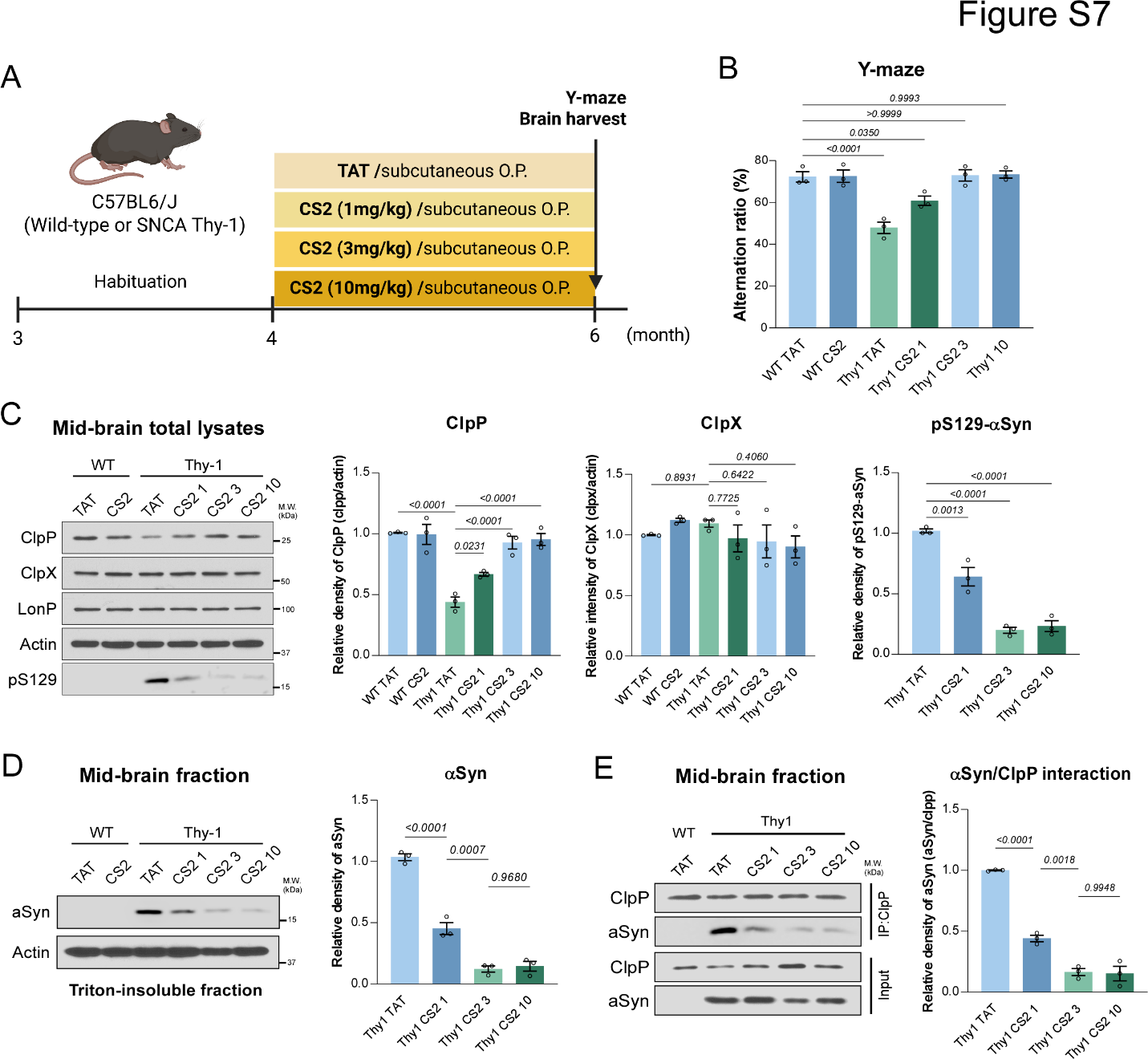
**

**Figure S7. Determine CS2 dosage for *in vivo* study.**

**(A)** Experimental schematic of TAT or CS2 administration *in vivo*: mThy1-hSNCA mice (Thy1) and their wild-type (WT) littermates were treated with TAT or CS2 with indicated doses (1 or 3 or 10 mg/kg/day) via subcutaneous osmotic pump (O.P.) (replaced every month) starting from age of 4-month till 6-month.

**(B)** Quantification of alternation ratio evaluated by Y-maze test in WT and Thy1 mice treated with TAT or CS2 peptide (n = 3 mice/group, one-way ANOVA with Tukey’s *post-hoc* test, data are mean ± SEM).

**(C)** Western blot and quantification of ClpP, ClpX and pS129-αSyn (pS129) in the midbrain of WT and Thy1 mice treated with TAT or CS2 (n = 3 mice/group, one-way ANOVA with Tukey’s *post-hoc* test, data are mean ± SEM).

**(D)** Western blot and quantification of αSyn in the insoluble fractions of the midbrain (n = 3 mice/group, one-way ANOVA with Tukey’s *post-hoc* test, data are mean ± SEM).

**(E)** Total protein lysates were harvested from the midbrain and subjected to immunoprecipitation using anti-ClpP antibody, followed by western blot analyses. Quantification of relative density of αSyn comparing to ClpP (pull-down) (n = 3 mice/group, one-way ANOVA with Tukey’s *post-hoc* test, data are mean ± SEM).

**
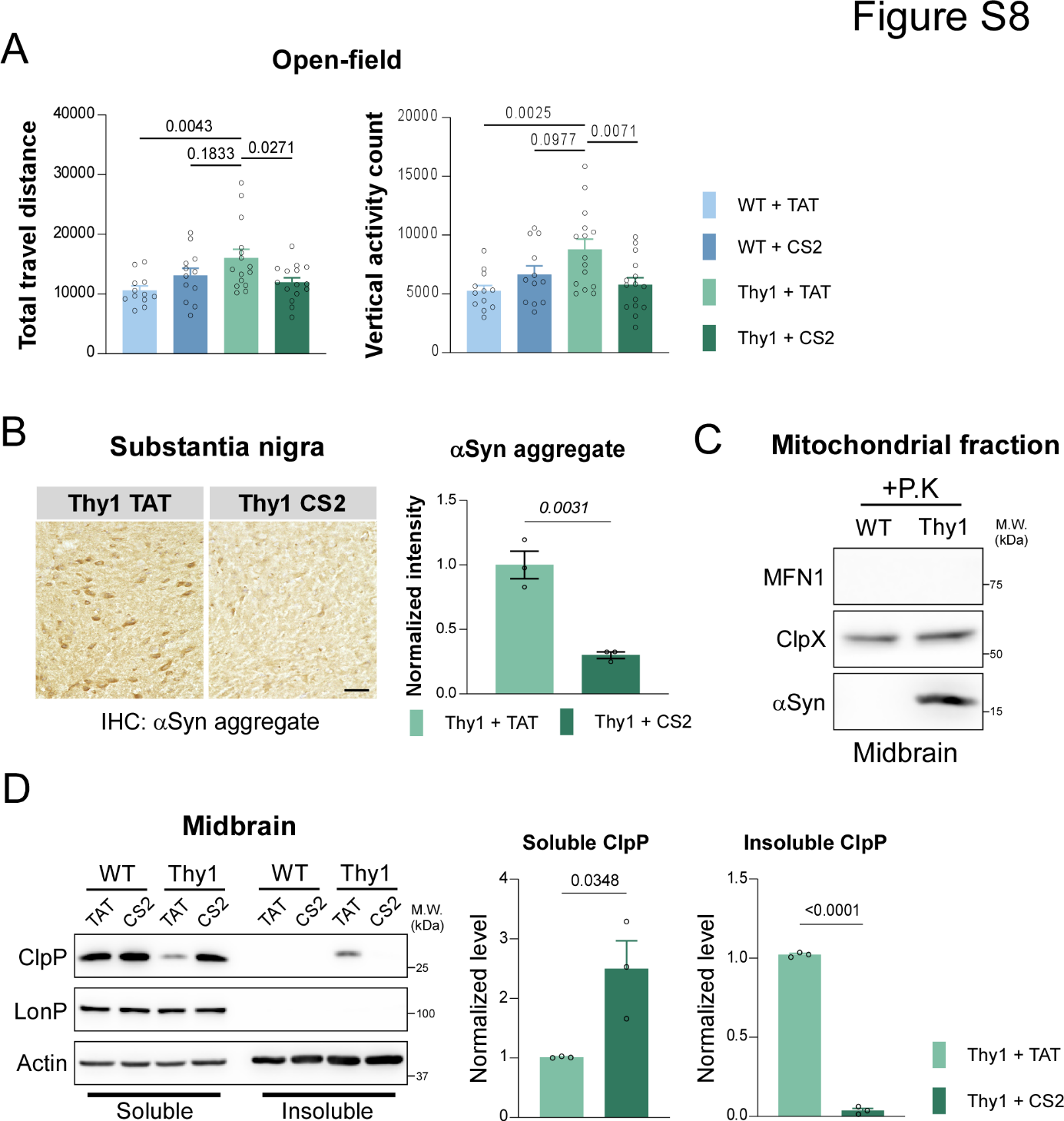
**

**Figure S8. CS2 treatment is protective in mThy1-hSNCA mouse model of PD.**

**(A)** Quantification of total travel distance and vertical activity of TAT or CS2 treated wild-type (WT) littermates and mThy1-hSNCA (Thy1) mice in the open-field chamber at age of 8-month (n = 12-15 mice/group, one-way ANOVA with Tukey’s *post-hoc* test, data are mean ± SEM).

**(B)** Representative images of αSyn aggregates staining (5G4) in substantia nigra of TAT or CS2 treated Thy1 mice (scale bar = 40µm). Quantification of the intensity of αSyn aggregates (n = 3 mice/group, one-way ANOVA with Tukey’s *post-hoc* test, data are mean ± SEM).

**(C)** Midbrain mitochondrial fractions were isolated from the WT and Thy1 mice, and subjected for western blot after proteinase-K digestion. Shown are the representative blots of 3 independent experiments.

**(D)** Digitonin-soluble and insoluble fractions were isolated from the midbrain of WT and Thy1 mice treated with TAT or CS2, and subjected for western blot. Shown are the representative blots of 3 independent experiments. Histogram summarizes normalized level of ClpP in the soluble and insoluble fractions (one-way ANOVA with Tukey’s post-hoc test, data are mean ± SEM).
