# Supplementary material for: Disrupting α-Synuclein–ClpP interaction restores mitochondrial function and attenuates neuropathology in Parkinson’s disease models
Source: Mol Neurodegener. 2025 Dec 22;20:126. doi: 10.1186/s13024-025-00918-w (PMC12751141; doi:10.1186/s13024-025-00918-w)

Supplementary file - uncropped gels and western blot images

Fig. 1A

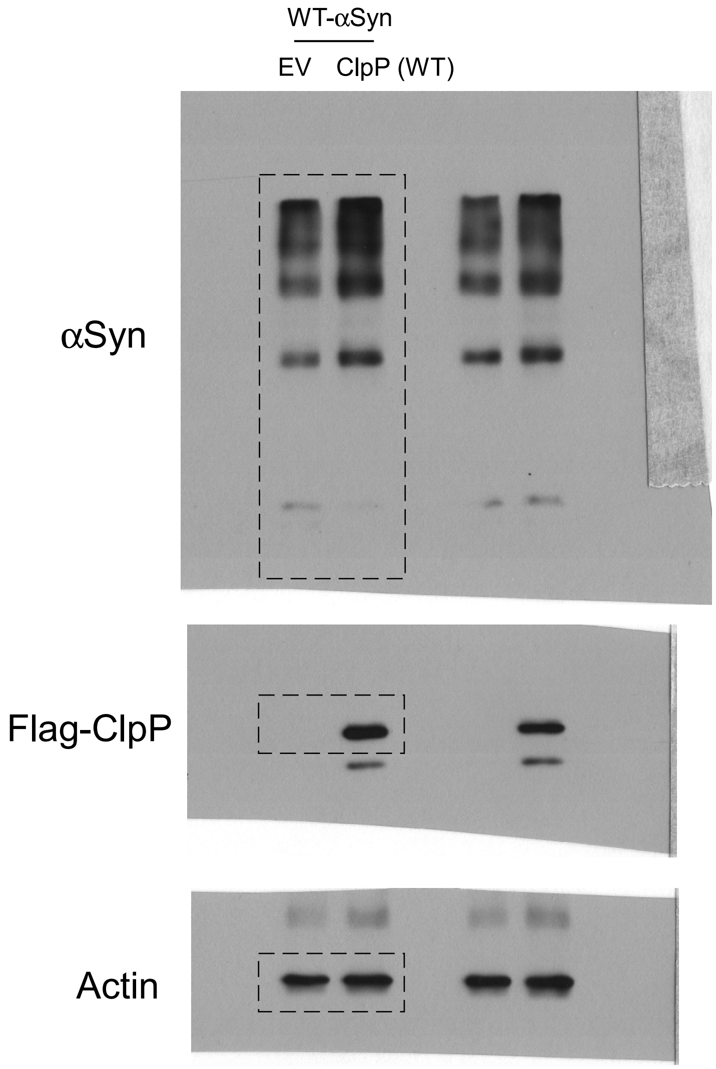

Fig. 1B

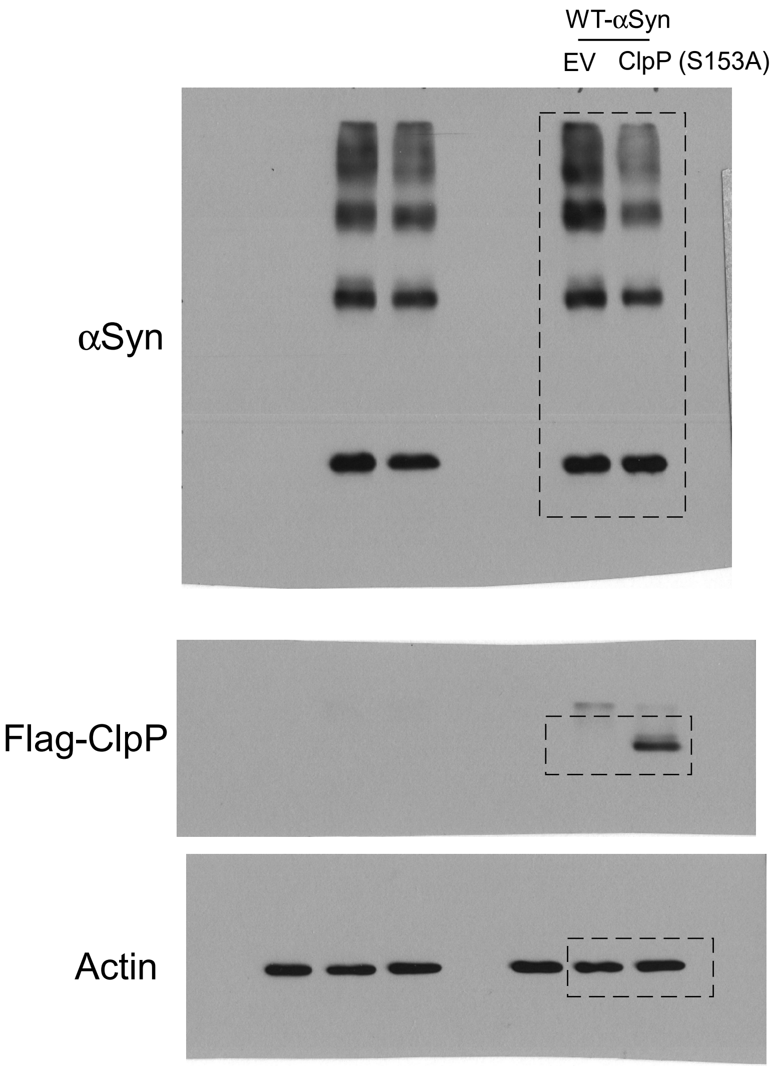

Fig. 2B

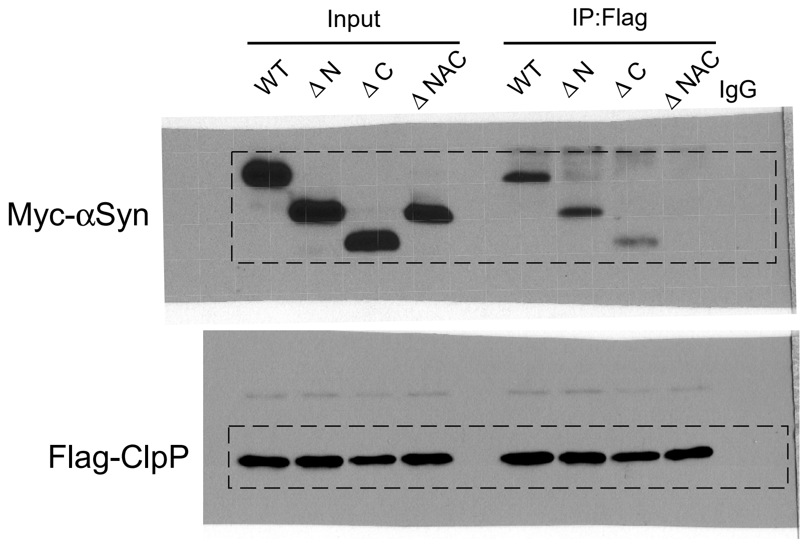

Fig. 2C

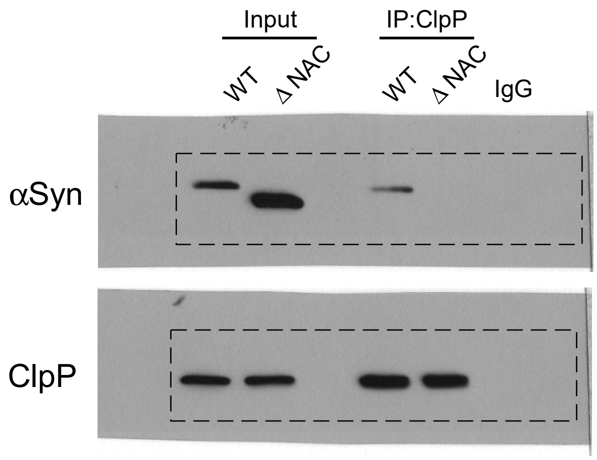

Fig. 2D

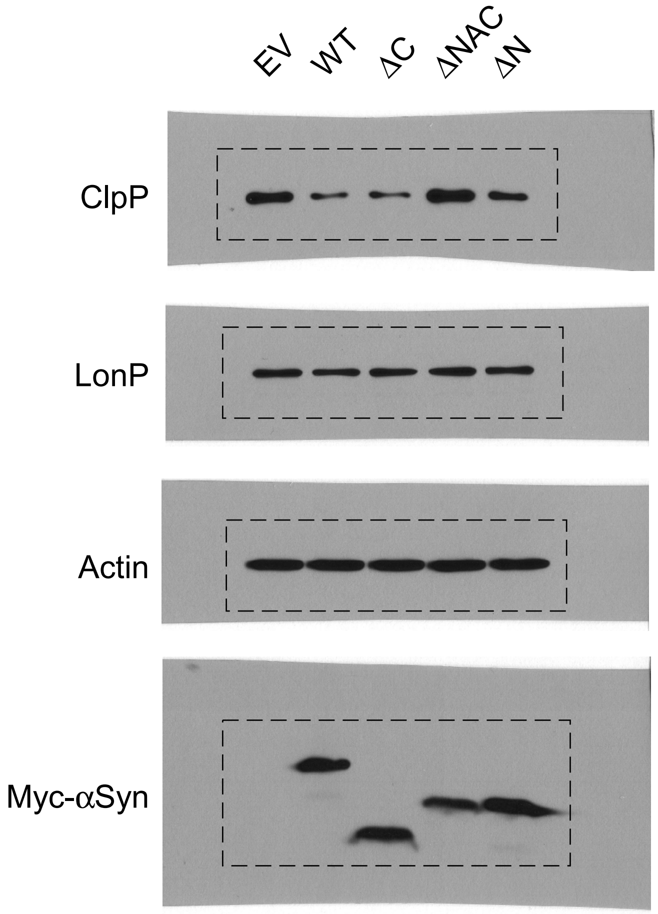

Fig. 3B

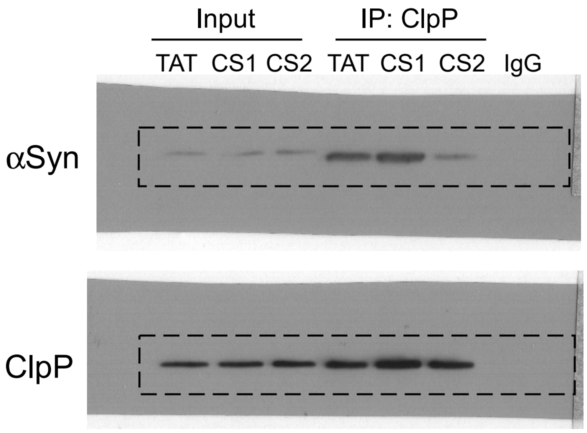

Fig. 3C

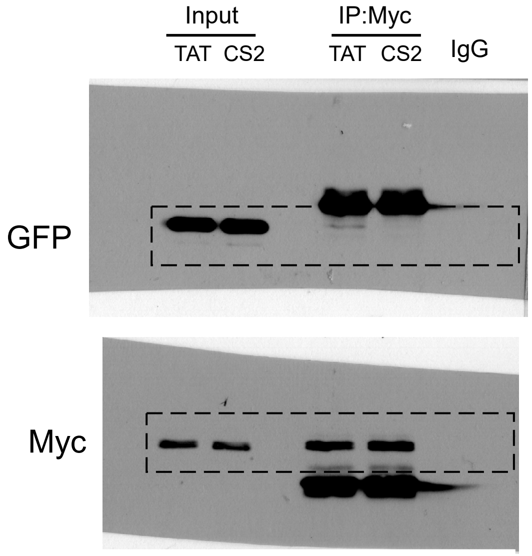

Fig. 3D

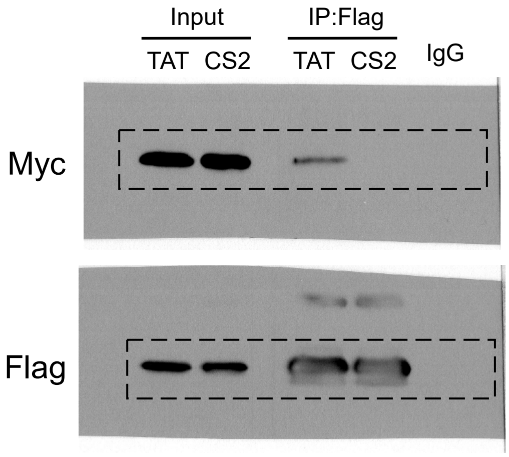

Fig. 3E

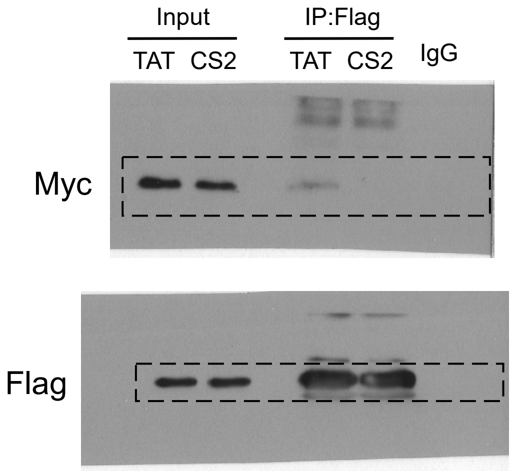

Fig. 3F

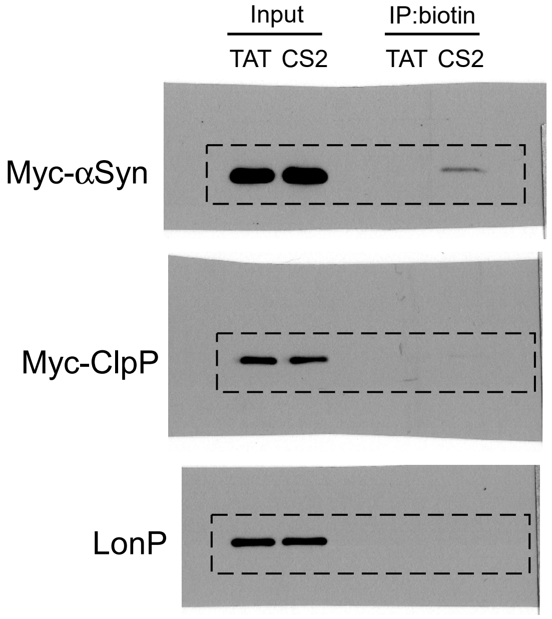

Fig. 4A

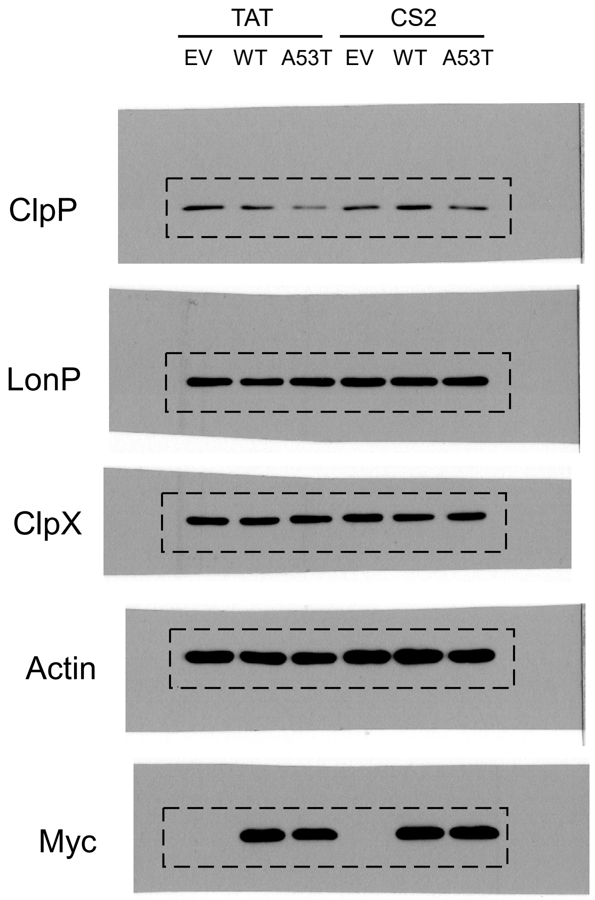
TAT
CS2
EV WT A53T EV WT A53T
ClpP
LonP
ClpX
Actin
Myc

Fig. 4B

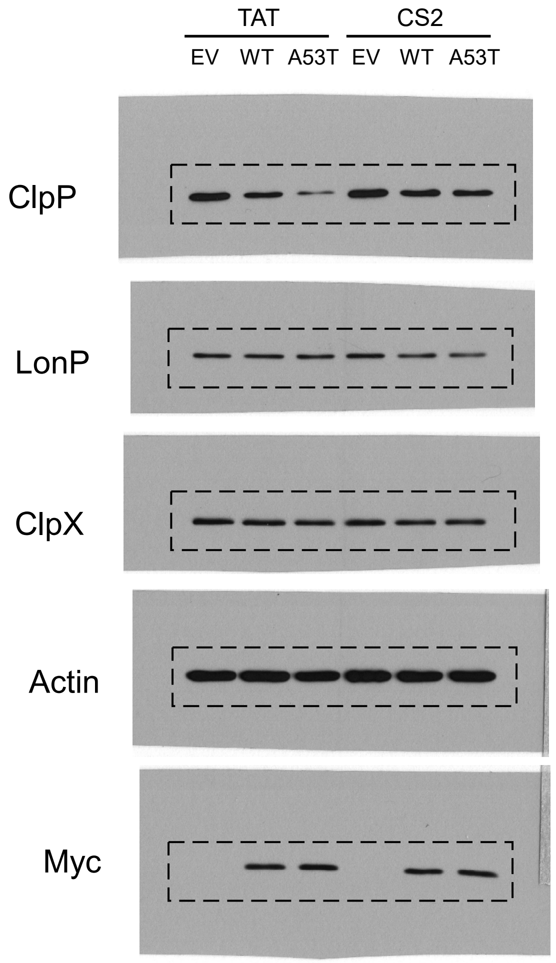
TAT
CS2
EV WT A53T EV WT A53T
ClpP
LonP
ClpX
Actin
Myc

Fig. 6C

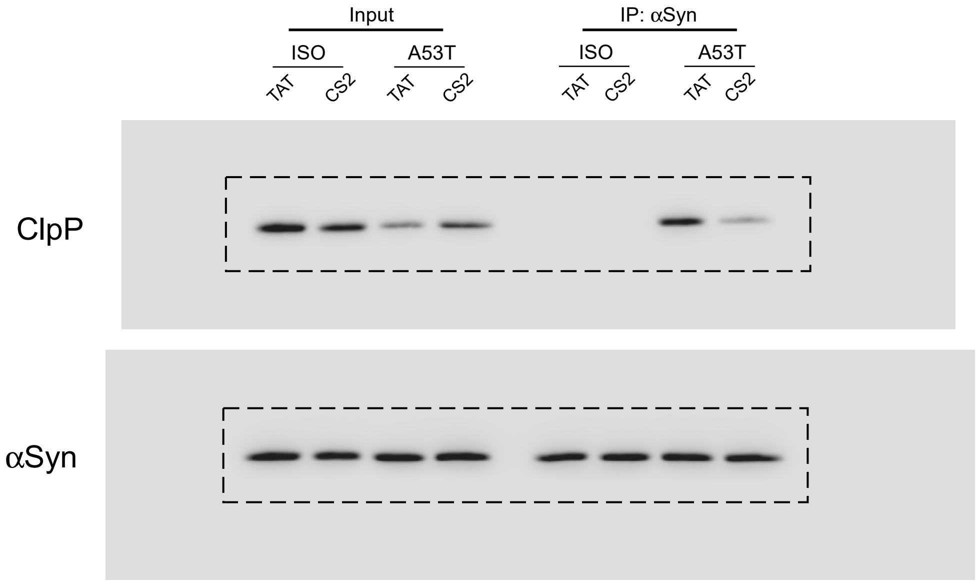
Input
IP: αSyn
ISO
A53T
ISO
A53T
TAT
CS2
TAT
CS2
TAT
CS2
TAT
CS2
ClpP
αSyn

Fig. 7E

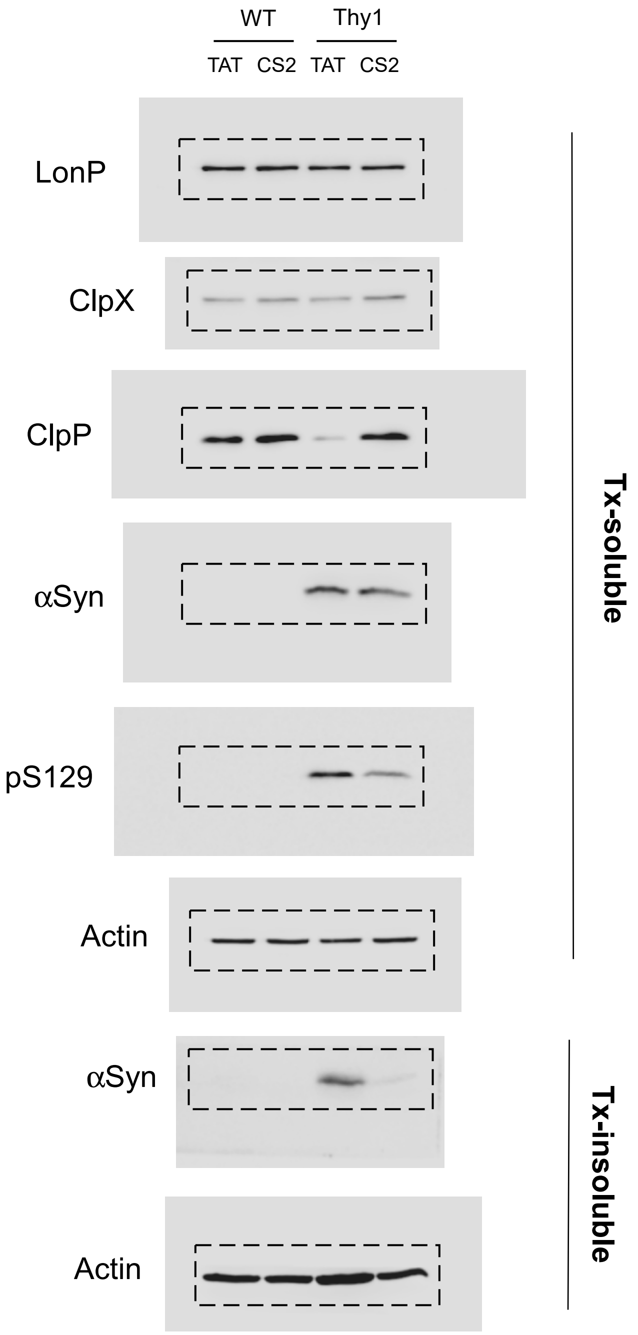
WT
Thy1
TAT
CS2
TAT
CS2
LonP
ClpX
ClpP
αSyn
pS129
Actin
αSyn
Actin
Tx-soluble
Tx-insoluble

Fig. S1A

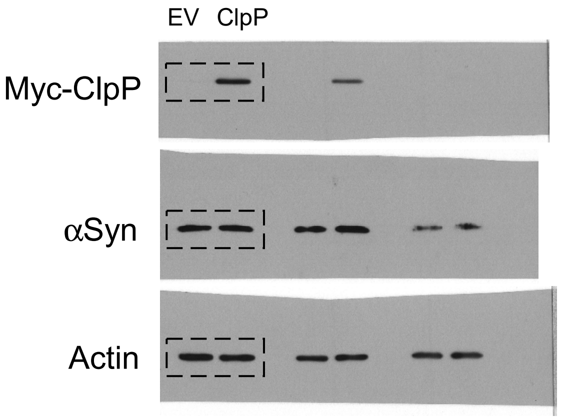

Fig. S1B

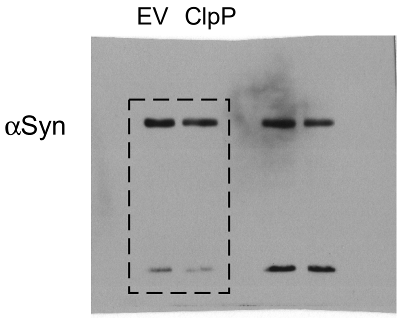

Fig. S1C

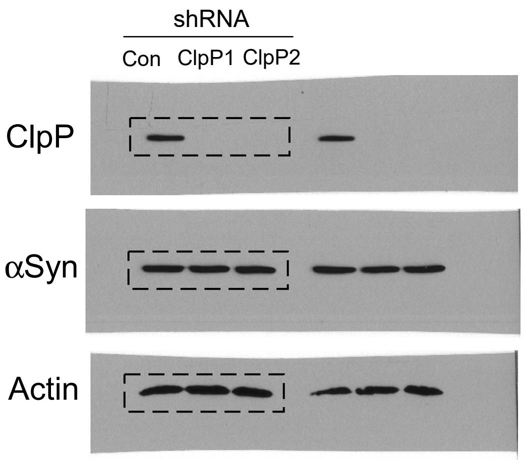

Fig. S1D

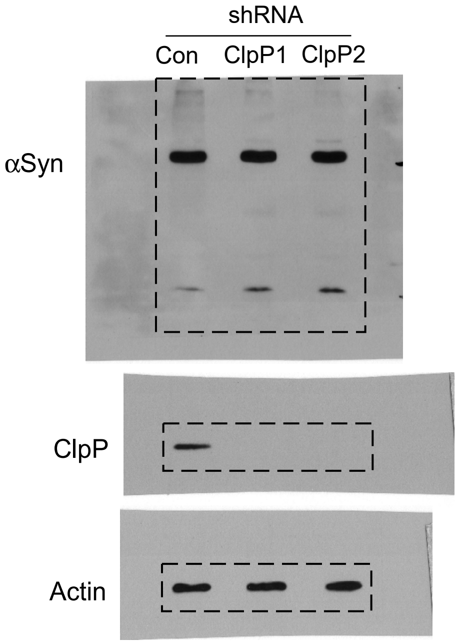

Fig. S1E

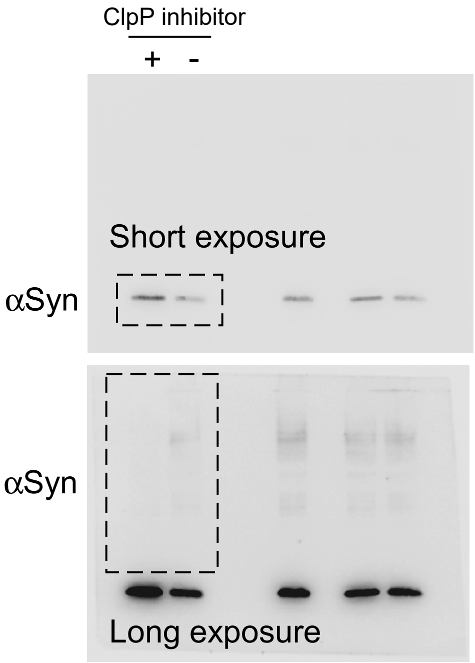

Fig. S1G

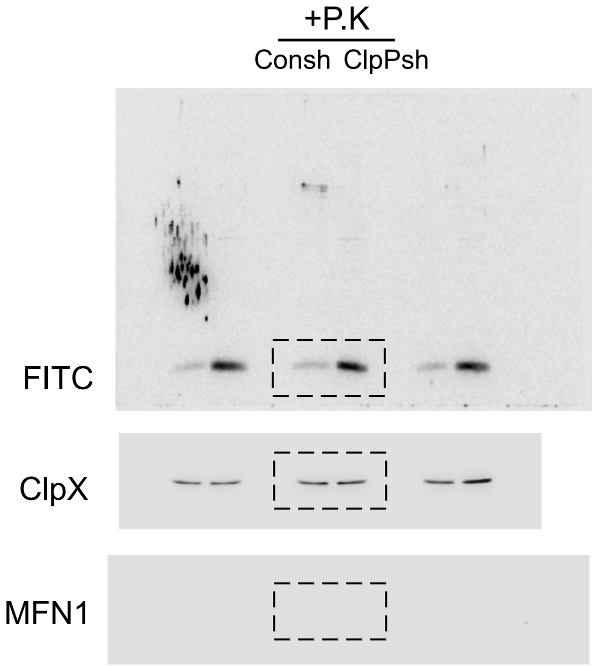

Fig. S2

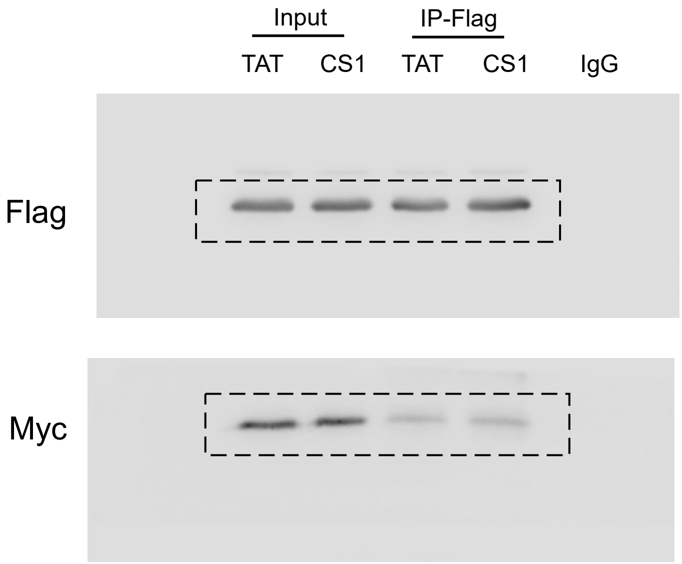

Fig. S3B

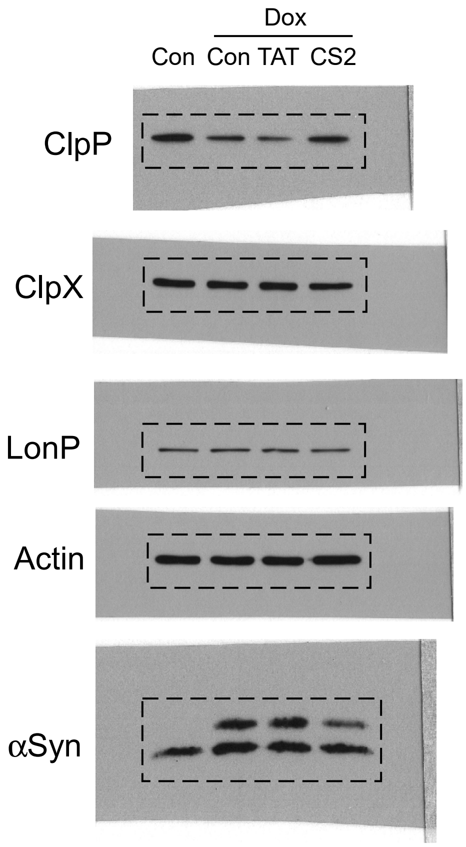

Fig. S4A

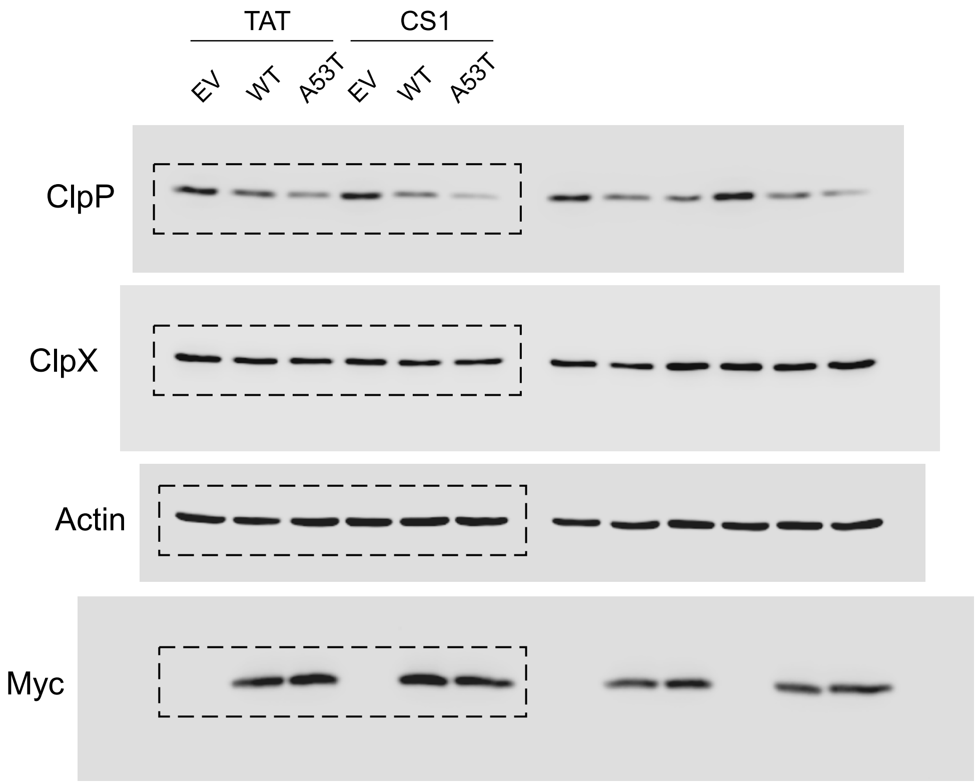

Fig. S4B

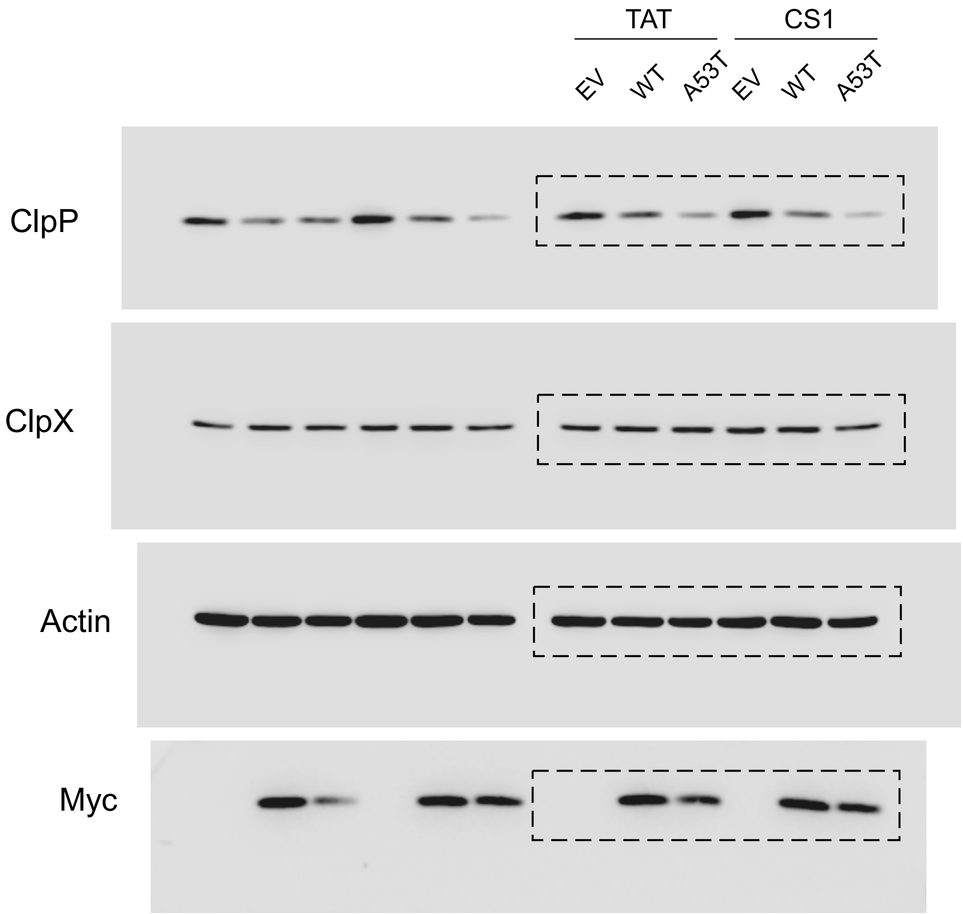

Fig. S5B

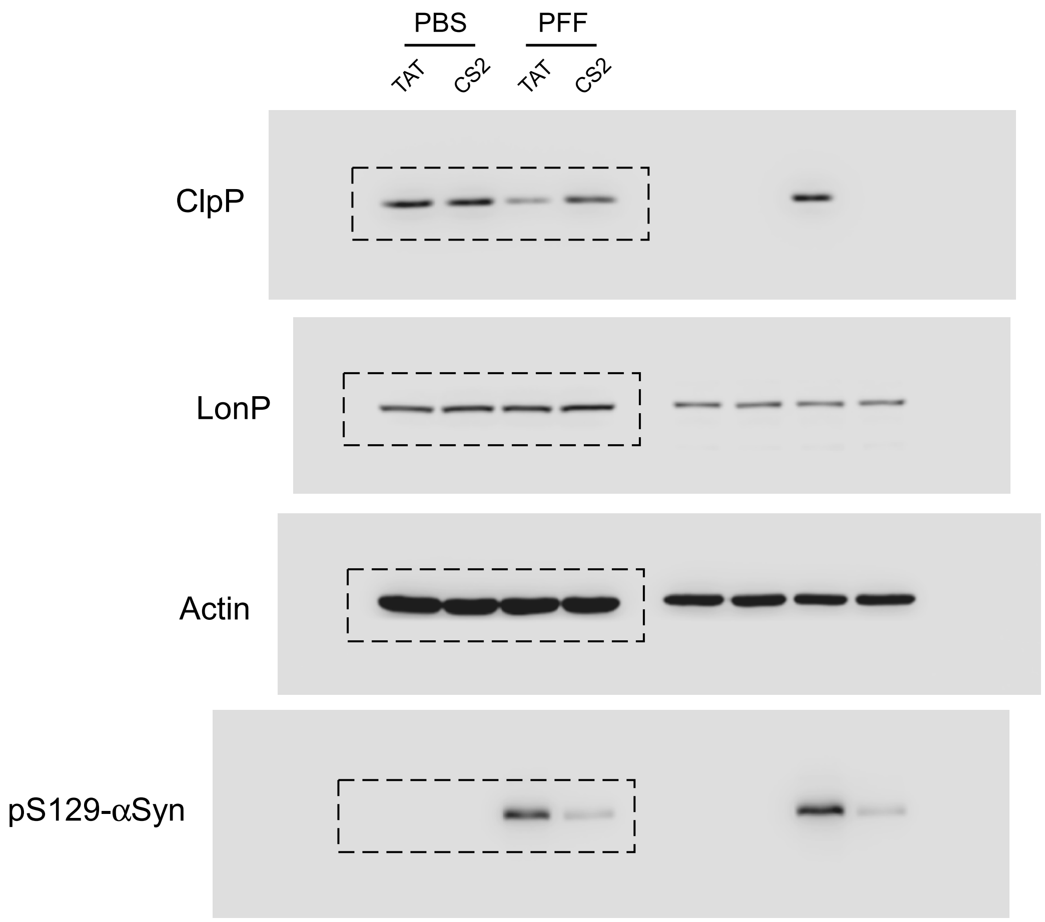

Fig. S6B

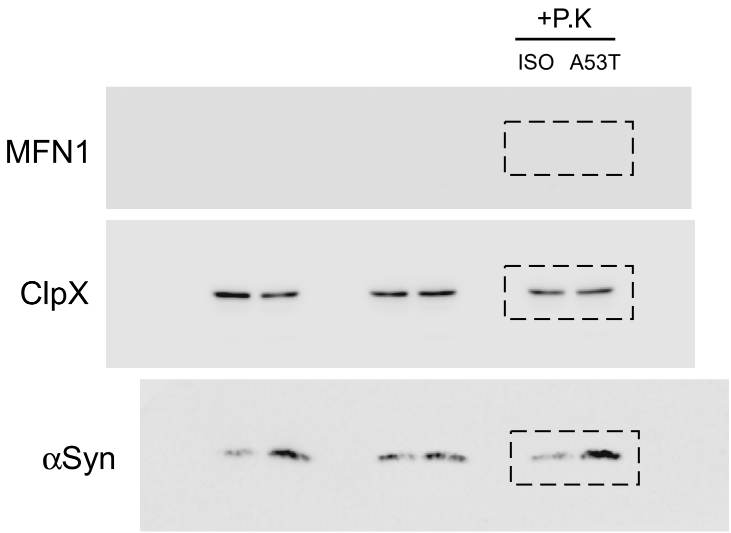

Fig. S6C

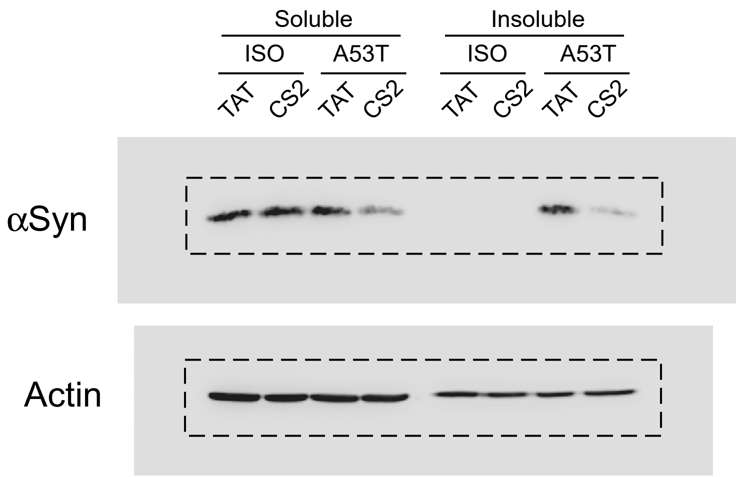

Fig. S7C

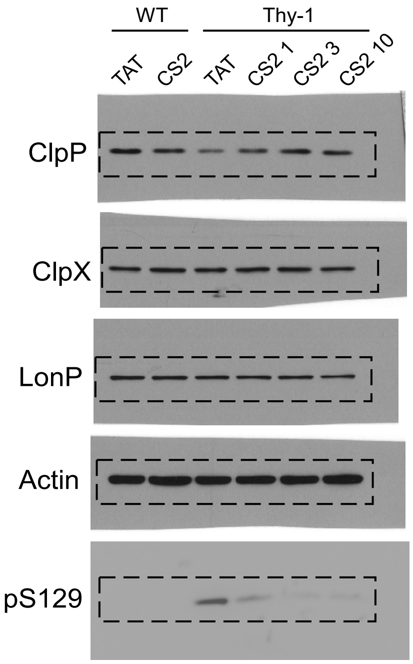

Fig. S7D

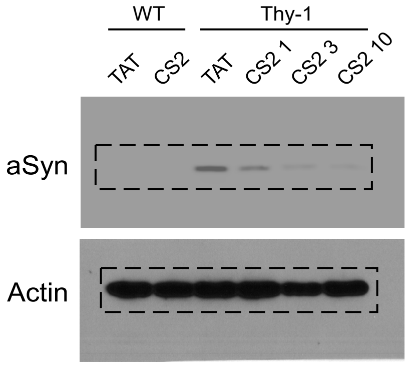

Fig. S7E

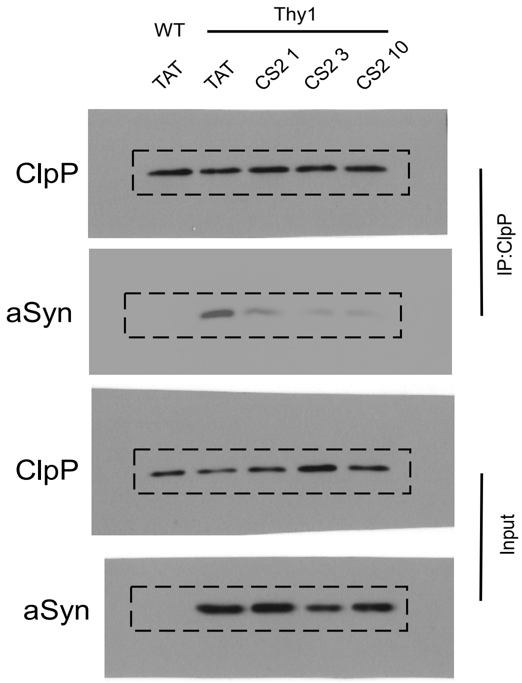

Fig. S8C

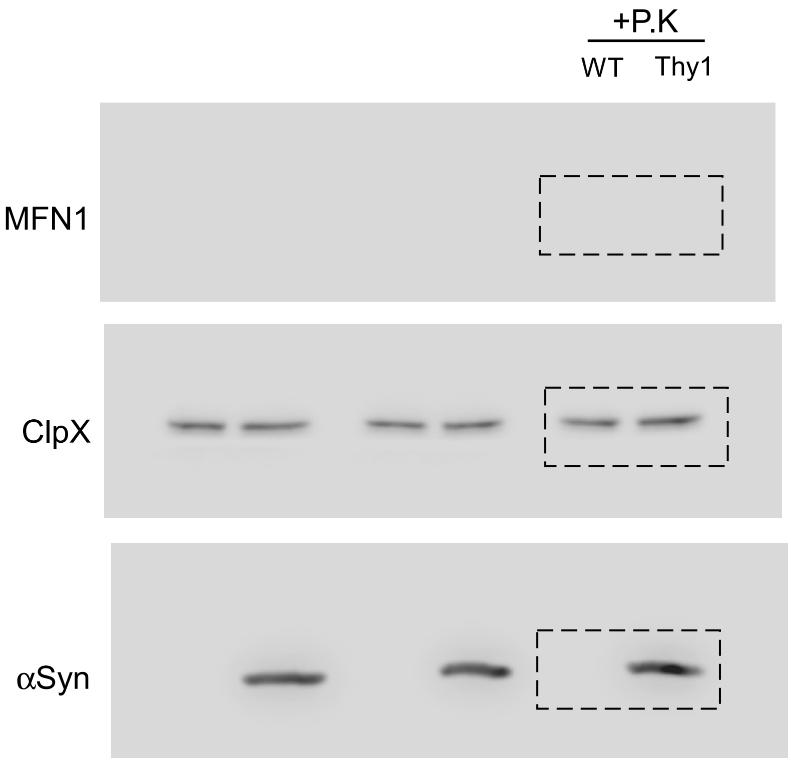

Fig. S8D

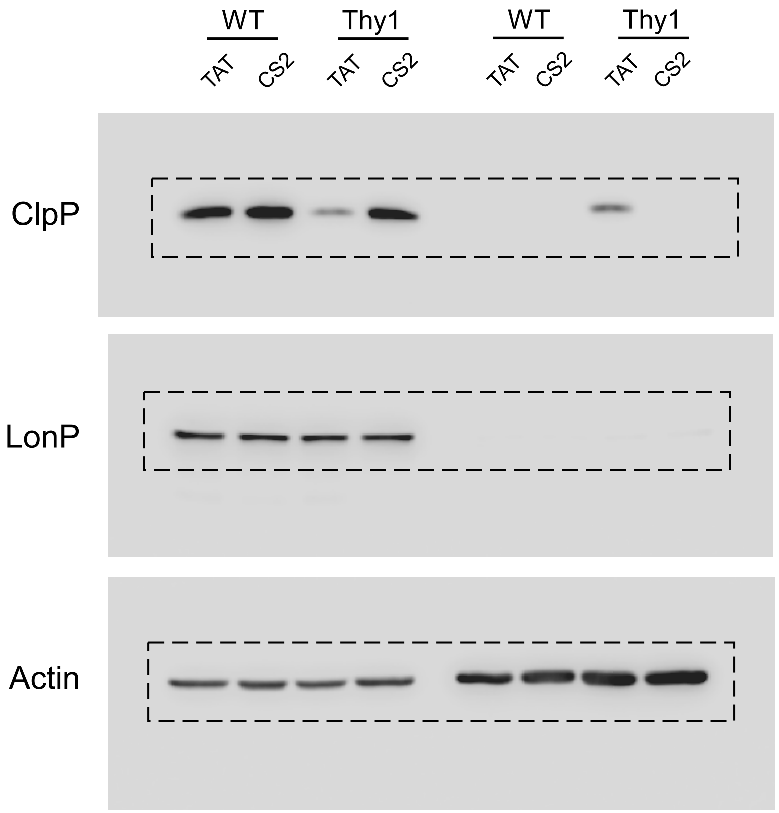

Supplement: Supplementary file 2 — Supplementary Material 2 [file 13024_2025_918_MOESM2_ESM.pdf]
